# Field irrigation using magnetized brackish water affects the growth and water consumption of *Haloxylon ammodendron* seedlings in an arid area

**DOI:** 10.3389/fpls.2022.929021

**Published:** 2022-08-25

**Authors:** Yi Guo, Quanjiu Wang, Xue Zhao, Zongyu Li, Mingjiang Li, Jihong Zhang, Kai Wei

**Affiliations:** ^1^State Key Laboratory of Eco-Hydraulic in Northwest Arid Region of China, Xi'an University of Technology, Xi'an, China; ^2^School of Water Resource and Hydropower, Xi'an University of Technology, Xi'an, China

**Keywords:** magnetized brackish water, *Haloxylon ammodendron*, soil salt distribution, water consumption, logistic growth function

## Abstract

Freshwater resources in arid areas are scarce, while there are abundant brackish water reserves that have great application potential for the irrigation of desert plants. However, brackish water irrigation will lead to soil salinization, which will inhibit plant growth. Magnetized water is a new technology that makes the use of brackish water feasible. The present study assessed the effects of irrigation using three water types (fresh, brackish, and magnetized brackish water) and five irrigation amounts (W1, 81 mm; W2, 108 mm; W3, 135 mm; W4, 162mm; and W5, 189 mm) on soil salinity and *Haloxylon ammodendron* seedling growth. Compared with fresh water, brackish water irrigation inhibited the growth of *H. ammodendron* and reduced water consumption. Irrigation with magnetized brackish water effectively improved the effect of soil salt leaching, promoted the growth and water absorption of *H. ammodendron* roots, and stimulated the growth of plant height, basal diameter, shoot length, and crown width. Based on the principal component analysis, the first three treatments of *H. ammodendron* comprehensive growth state were FW4, FW3, and MBW4, respectively. This showed that magnetized brackish water combined with an appropriate irrigation amount was helpful to optimize the growth of *H. ammodendron* seedlings on the basis of fresh water saving. Therefore, magnetized brackish water irrigation is an effective strategy for ensuring the establishment and growth of *H. ammodendron* seedlings in arid and water-deficient areas.

## Introduction

Xinjiang is located in the arid inland area of Northwest China, where evaporation is strong and rainfall is scarce (Danierhan et al., 2013; Wang Z. et al., 2019; Zhang et al., 2022). Its unique climate characteristics have created a serious shortage in regional water resources, and the discrepancy between water supply and demand has become large. With population growth and economic development, crop planting areas continue to expand and agricultural water used for oasis planting has increased annually. Agricultural water accounted for 86.99% of all water used in Xinjiang in 2020 (China Water Resources Bulletin, 2020). Fresh water resources are mainly used to irrigate crops such as cotton, wheat, and corn. However, it is difficult to meet the normal water supply demands during the peak water demand period, and the ecological water required by shelter forests and desert plants is not always guaranteed (Chen et al., 2015; Zhang et al., 2016, 2017, 2019; Wang et al., 2018; He et al., 2022; Hou et al., 2022). Shallow brackish water, widely distributed in Xinjiang, is a water resource, which has huge agricultural irrigation potential that could make up for the shortage of available fresh water (Wang et al., 2011; Chen et al., 2018; Ma et al., 2022). Thus, brackish water resources may be used to alleviate the discrepancy between agricultural and ecological water demands and supply.

To date, research on the use of brackish water for irrigation has mostly focused on agricultural crops such as wheat, cotton, corn, and rice, and has generally focused on variables such as crop growth, yield, water use efficiency, and physical and chemical properties of the soil (Deb et al., 2016; Tan et al., 2017; Huang et al., 2019; Liu et al., 2019; Chen et al., 2020; Zhu et al., 2021). Few studies have examined the use of brackish water to irrigate desert plants. Some studies have observed that, despite the low water quality requirements for desert plants, salt accumulation caused by brackish water irrigation still suppresses plant growth and can even lead to plant death (Wang et al., 2010; Chen et al., 2011; Zhang et al., 2020). Therefore, the key to successfully using brackish water is alleviating the damage to plants caused by salt accumulation in soil.

In recent years, the development of magnetized water technology has provided a new solution to a series of problems such as secondary soil salinization and soil structural changes caused by brackish water irrigation. Magnetized water technology involves flowing of water vertically or horizontally at a certain speed through a magnetic induction line generated by a magnetic field of a specific strength (Zhao et al., 2021). Under the action of the magnetic field, the average distance between water molecules increases, and the hydrogen bonds are weakened or even broken, causing the large clusters of associated water molecules to become smaller as free monomer molecules and dimer molecules (Amiri and Dadkhah, 2006; Toledo et al., 2008). Relevant studies have shown that, compared with the physical and chemical properties of non-magnetized water, the chemical bond angle and water-ion colloidal radius of magnetized water decrease, the osmotic pressure and solubility increase, the viscosity coefficient and surface tension of water decrease, the pH value of water increases, and the amount of dissolved oxygen in the water increases (Shimokawa, 2004; Wang Q. J. et al., 2019). To date, magnetization treatment has been thought to enhance salt leaching from the soil, which thereby reduces the damage caused by brackish water or saline water irrigation to the plants. For example, in a study by Bu et al. (2010), the desalination rate of non-magnetized water to soil was 10–20%, while that of magnetized water was 20–30%. Zhou et al. (2021) demonstrated that magnetized water irrigation could increase salt leaching from the soil and reduce the soil salt contents in the salinized soil. Mostafazadeh-Fard et al. (2011) observed that magnetized water can effectively reduce ${\text{SO}}_{4}^{2-}$ ion content in soil and improve plant survival rate. Liu X. M. et al. (2020) also observed that magnetization treatment alleviated the inhibitory effect of soil salinity on grape growth. In summary, the above applications of magnetized water have focused on crop irrigation.

*Haloxylon ammodendron* is a perennial shrub that is widely distributed in the desert regions of Central Asia and the arid and semi-desert regions of Northwest China (Liu et al., 2011; Buras et al., 2012; Cui et al., 2017; Lü et al., 2019, 2022; Zhou et al., 2022). *H. ammodendron* is planted over an area of 114,000 km^2^ in China, mainly distributed in the Xinjiang Junggar Basin, Tarim Basin, the Alxa Desert in Inner Mongolia, Qinghai Qaidam Basin, and Gansu Hexi Corridor (Hong et al., 2019). The Xinjiang *H. ammodendron* area accounts for 68.1% of the national *H. ammodendron* desert area (Song et al., 2021). Since *H. ammodendron* has high ecological adaptability, with drought resistance, cold resistance, high temperature resistance, salt-alkali resistance, and wind erosion resistance, it is the species with the highest biomass in the desert ecological zone (Tobe et al., 2000; Ma et al., 2007; Zhuang and Zhao, 2017; Yang et al., 2020). *H. ammodendron* is a strong pioneer plant species that has windbreak and sand fixation functions and plays a key role in the stability of the northwest desert and semi-desert ecological zone (Huang et al., 2003; Sheng et al., 2005; Dai et al., 2022; Li et al., 2022; Shi et al., 2022). In addition, *H. ammodendron* is utilized as a fuel species, pasture resource, and substrate for the precious parasitic medicinal plant *Cistanche deserticola* (Thevs et al., 2013; Yang et al., 2014; Shao et al., 2022). However, over the past few decades, under the influence of human activities and climate change, the population of *H. ammodendron* has decreased significantly (Zhang and Chen, 2002). Moreover, during the seed germination season, inconsistent rainfall has damaged the seedling supplementation of the *H. ammodendron* population, and as a large number of seedlings have died, the age structure of the *H. ammodendron* population has generally shifted, with the community exhibiting characteristics of retrograde succession (Huang et al., 2008). The settlement and growth of seedlings in a community is an important process that determines whether the population can be regenerated naturally (Liu et al., 2012). Given the important role of *H. ammodendron* in desert ecosystems, it is necessary to study the growth and development of *H. ammodendron* seedlings.

In recent years, studies have examined the growth of *H. ammodendron* seedlings under different irrigation amounts with different irrigation water qualities (Shan et al., 2008, 2009; Liu et al., 2012, 2022; Zhang et al., 2020). These studies have mainly focused on the physiological and ecological response characteristics of *H. ammodendron*, and a limited number of studies have analyzed the soil salt distribution, water consumption, and biomass water use efficiency. Furthermore, the effect of magnetized brackish water irrigation on the growth of *H. ammodendron* seedlings has not been clarified. Thus, the objectives of the study were to (1) evaluate the effects of different irrigation water types and amounts on soil salt distribution, (2) quantitatively analyze the effects of different irrigation types and amounts on the growth of *H. ammodendron* seedlings, and (3) determine the effects of different irrigation water types and amounts on water consumption and biomass water use efficiency.

## Materials and methods

### Site description

This experiment was conducted at the Bazhou Irrigation Experimental station (41°35′N, 86°10′E, 901 m above sea level), located near the edge of the oasis in Korla City on the northern margin of the Taklimakan Desert in Xinjiang Autonomous Region. The region has a temperate continental arid desert climate, with hot summers and cold winters, and is generally dry with little rain. The annual temperature is 8°C. The total precipitation at the experimental site during the *H. ammodendron* growth season was 55.9 mm. In spring, sandstorm activities are intense, with an average wind speed of 2.4 m/s and a maximum wind speed of 22 m/s. The soil at the site is sandy (sand 87.3%, silt 11.6%, and 1.1% cay) (USDA texture class) with little organic matter and limited mineral nutrients. The local plant community is dominated by *H. ammodendron, Tamarix chinensis*, and *Calligonum mongolicum*.

The fresh water and brackish water used for irrigation were taken from the Kongqi River and local groundwater, respectively. The electrical conductivity (EC) and main elemental contents of the brackish and freshwater are shown in Table 1.

**Table 1:** Electrical conductivity (EC) and major ion contents of brackish and fresh water.

**Water type**	**EC (μS cm^−1^)**	**Major cation (mg l** ^**−1**^ **)**			**Major anion (mg l** ^**−1**^ **)**			
		**Na^+^+K^+^**	**Mg^2+^**	**Ca^2+^**	**HCO^3−^**	**CO^2−^**	**Cl^−^**	** ${\text{SO}}_{4}^{2-}$ **
Brackish water	3027.42a	281.0a	75.9a	118.5a	386.1a	21.12a	249.0a	473.8a
Fresh water	953.25b	89.49b	34.79b	56.17b	205.55b	15.59b	97.6b	145.87b

Different letters within a column indicate significant differences between water types at p < 0.05.

### Experimental design

The field experiment consisted of a completely randomized factorial combination of three kinds of irrigation water (fresh water, magnetized brackish water, and brackish water; F, MB, and B, respectively) and five water supply levels (81, 108, 135, 162, and 189 mm; W1, W2, W3, W4, and W5, respectively). The water supply was designed to be according to the relevant literature (Liu et al., 2018) and the actual production experience of local farmers. In total, there were 15 treatments (FW1, FW2, FW3, FW4, FW5, MBW1, MBW2, MBW3, MBW4, MBW5, BW1, BW2, BW3, BW4, and BW5) with three replicates per treatment, making a total of 45 plots. Each plot was 1.8 m × 1.8 m and the adjacent plots were separated by partitions buried to a depth of 1.8 m to eliminate the lateral movement of soil water. The planted *H. ammodendron* were spaced 0.6 m apart. The *H. ammodendron* seedlings were planted on April 24, 2021.

Brackish water was magnetized by passing through a magnetic field at a specified speed. The magnetized water device consisted of a water pipeline, water box, peristaltic pump, and permanent magnet. The permanent magnet, with a magnetic field intensity of 3000G, was mounted on the outer wall of the water pipeline. When the brackish water passed through the pipe, it was magnetized.

### Measurements and calculations

#### Growth indicators

The growth indicators were measured throughout the growing season. From May to September, the plant height, basal diameter, new shoot length, and crown width were measured at the end of each month. The roots of *H. ammodendron* were excavated in late September to measure the vertical root length, and then the aboveground portion of *H. ammodendron* and the washed roots were weighed after drying in the oven (at 80°C for 48 h) until a constant weight was obtained. The root shoot ratio was calculated by the following formula:

(1) $$\begin{array}{r} Root\;shoot\;ratio=\frac{Root\;dry\;weight}{Shoot\;dry\;weight} \end{array}$$

#### Soil moisture content and soil salt content

Soil samples were collected during the middle days of each month. Soil samples were collected at 10 cm intervals from 0 to 40 cm and at a 20 cm interval from 40 to 100 cm. Soil moisture content was determined using the drying method. After the soil was dried and crushed, soil salinity was determined using a DDS-307A conductivity meter (Shanghai Precision & Scientific Instrument Inc., Shanghai, China) with a soil-to-water ratio of 1:5.

#### Description of the logistic function

The logistic function was originally proposed by ecologists to describe the laws of biological population growth and has subsequently been used in modeling plant height growth, leaf area expansion, and grain filling (Zahedi and Jenner, 2003; Bagheri et al., 2014; Liu Y. H. et al., 2020). The logistic growth function was used here to analyze the change in plant height, basal diameter, new shoot length, and crown width:

(2) $$\begin{array}{r} H=\frac{H_{m}}{1+a_{1}*e^{a_{2}*t}} \end{array}$$

(3) $$\begin{array}{r} D=\frac{D_{m}}{1+b_{1}*e^{b_{2}*t}} \end{array}$$

(4) $$\begin{array}{r} S=\frac{S_{m}}{1+c_{1}*e^{c_{2}*t}} \end{array}$$

(5) $$\begin{array}{r} C=\frac{C_{m}}{1+d_{1}*e^{d_{2}*t}} \end{array}$$

where, H, D, S, and C represent plant height (cm), basal diameter (mm), new shoot length (cm), and crown width (cm^2^) respectively; *H*_*m*_, *D*_*m*_, *S*_*m*_, and *C*_*m*_ represent maximum plant height (cm), maximum basal diameter (mm), maximum new shoot length (cm), and maximum crown width (cm^2^), respectively; *a*_1_, *a*_2_, *b*_1_, *b*_2_, *c*_1_, *c*_2_, *d*_1_, and *d*_2_ are empirical parameters, and t is the number of days after planting (day).

#### Salt accumulation calculation

The salt balance formula was as follows (Zhou et al., 2021):

(6) $$\begin{array}{r} \Delta S=S_{2}-S_{1} \end{array}$$

where *S*_1_ is the initial soil salinity, *g*; and *S*_2_ is the final soil salinity, *g*.

Soil salt accumulation or desalination rate was calculated as follows:

(7) $$\begin{array}{r} G=\frac{S_{2}-S_{1}}{S_{1}}*100\% \end{array}$$

where *G* is accumulation or desalination rate, %. When *G* is positive, it indicates that the soil is in a salt accumulation state; and if *G* is negative, it indicates that the soil is in a desalination state.

#### Evapotranspiration and biomass water use efficiency calculation

Evapotranspiration (water consumption) was calculated based on the water-balance equation:

(8) $$\begin{array}{r} ET=P+U+\Delta W+I-D-R \end{array}$$

where P is rainfall (mm); U is groundwater recharge (mm); Δ*W* is the change in water storage in the 1-m soil profile (mm); I is irrigation amount (mm); D is the deep percolation (mm); R is the surface runoff (mm). The effect of groundwater was negligible due to the depth of the groundwater table (>7 m). Since there was no surface runoff during the experiment, this value was also ignored.

*D* was calculated using the following equation (Tan et al., 2017):

(9) $$\begin{array}{r} D=P+I+SWS-1000\theta_{FC} \end{array}$$

where SWS is soil water storage in the 1-m soil profile one day before irrigation (mm); and θ_*FC*_ is the field water holding capacity (cm^3^/cm^3^).

Biomass water use efficiency was calculated following (Liao et al., 2022):

(10) $$\begin{array}{r} WUE_{B}=\frac{B}{ET} \end{array}$$

where *B* is biomass of *H. ammodendron*, kg/hm^2^.

### Data analysis

The Excel software was used for calculations and the SPSS 22.0 (IBM Crop., Armonk, New York, NY, USA) software was used for statistical analysis and principal component analysis (PCA). The growth indexes, soil salinity, water consumption, and biomass water use efficiency were analyzed for average and standard deviation for each treatment (*n* = 3). Differences were determined using Duncan's multiple range test at the 5% level of significance. OriginPro 2019 (Origin Lab Corporation, Northampton, MA, USA) was used to create the graphs.

The determination coefficient (*R*^2^) and the normalized root mean square error (nRMSE) were used to evaluate the accuracy of the growth functions. These statistical indexes were calculated as follows:

(11) $$\begin{array}{r} R^{2}=1-\frac{\sum_{i=1}^{n}{\left(M_{i}-S_{i}\right)}^{2}}{\sum_{i=1}^{n}{\left(M_{i}-\bar{M}\right)}^{2}} \end{array}$$

(12) $$\begin{array}{r} nRMSE=\frac{1}{\bar{M}}\sqrt{\frac{\sum_{i=1}^{n}{\left(S_{i}-M_{i}\right)}^{2}}{n}}*100 \end{array}$$

where *M*_*i*_ is the measured value; *S*_*i*_ is the simulated value; $\bar{M}$ is the average value of the measured data; and n is the number of measurements.

## Results

### Soil salt distribution

Based on the salt balance theory, the change in soil salt reserves of the 0–100 cm root layer with different irrigation treatments in 1 m^2^ unit during the growing season was calculated. The changes in soil salt were significantly different (*p* < 0.05) among all treatments during the growing season (Table 2). Compared with the initial soil salt content in April, the soil salt reserves significantly decreased in May in all treatments, and all the soils were in desalination states. Looking at the soil desalination rates in May, within each respective irrigation type, the soil desalination rate increased with increasing irrigation amount, peaking with the W5 irrigation amount at 34.15, 27.68, and 22.05% for the F, MB, and B treatments, respectively. Within the same irrigation amount, the soil desalination effects of the three water types were ordered F > MB > B. Relative to the desalination effect of freshwater irrigation, with irrigation amounts of W1, W2, W3, W4, and W5, the desalination rates of magnetized brackish water irrigation were lower by 22.27, 23.23, 11.83, 22.67, and 18.95%, while those of brackish water irrigation was lower by 51.22, 34.48, 35.08, 34.38, and 35.43%, respectively. Further comparisons of the soil salt reserves of treatments showed that the soil was in the salt accumulation state in September. Within each respective irrigation type, soil salt accumulation rate increased with increasing irrigation amount, and peaked in the W5 irrigation treatment at 19.68, 25.21, and 21.72% in the F, MB, and B treatments, respectively. Within the same irrigation amounts, the soil salt accumulation rate of different treatments was ordered MB > B > F. Compared with fresh water irrigation, under the W1, W2, W3, W4, and W5 irrigation amounts, the salt accumulation rates of magnetized brackish water irrigation were higher by 143.52, 69.98, 46.55, 39.85, and 28.10%, while those of brackish water irrigation was higher by 7.43, 31.81, 23.69, 14.10, and 10.37%, respectively.

**Table 2:** Soil salt distribution for all irrigation treatments in the 100 cm soil profile.

**Treatment**	**Initial soil salt amount (g/m^2^)**	**Soil salt amount in May (g/m^2^)**	**Difference in soil salt amount (g/m^2^)**	**Salt desalination rate**	**Soil salt amount in Sept. (g/m^2^)**	**Difference in soil salt amount (g/m^2^)**	**Salt accumulation rate**
FW1	11030.30efg	8989.16bc	−2041.14cd	17.96	11492.40ghi	462.1j	4.71
MBW1	11031.52efg	9478.45ab	−1553.07b	13.96	12235.24fg	1203.72h	11.47
BW1	10603.12efg	9616.74ab	−986.37a	8.76	11083.53hi	480.41j	5.06
FW2	10077.56gh	7683.81e	−2393.75ef	23.29	10925.42i	847.86i	8.96
MBW2	11266.26def	9150.40bc	−2115.86de	17.88	12917.72ef	1651.46g	15.23
BW2	11310.24def	9498.35ab	−1811.89bc	15.26	12583.61	1273.37h	11.81
FW3	9340.71hi	6787.41f	−2553.30f	26.54	10518.59i	1177.88h	13.17
MBW3	8980.37i	6830.69f	−2149.68de	23.4	10659.95i	1679.58g	19.3
BW3	10395.46fg	8503.62cd	−1891.83cd	17.24	12029.00fge	1633.54g	16.29
FW4	12168.78bcd	8333.28cde	−3835.50i	31.27	14177.53cd	2008.75f	17.09
MBW4	13124.18b	9761.58ab	−2384.35ef	24.18	16181.02b	3056.84b	23.9
BW4	11512.96cde	9128.62bc	−3362.59h	20.52	13688.72de	2175.75e	19.5
FW5	12346.97bc	8119.30de	−4227.67j	34.15	14703.24c	2356.27d	19.68
MBW5	14341.69a	10278.66a	−4063.03ij	27.68	17868.59a	3526.90a	25.21
BW5	13139.85b	10170.78a	−2969.07g	22.05	15914.35b	2774.51c	21.72
**Significance**							
Irrigation type	^**^	^**^	^**^		^**^	^**^	
Irrigation							
amount	^**^	^**^	^**^		^**^	^**^	

The data are all average (the number of samples for each treatment is 3) in the table. Differences were determined using Duncan's multiple range test. Different letters within a column indicate significant differences among treatments at p < 0.05.

** Indicates that it is significant at level of 0.01.

### Aboveground biomass, belowground biomass, root to shoot ratio, and root length

**Figure 1:**
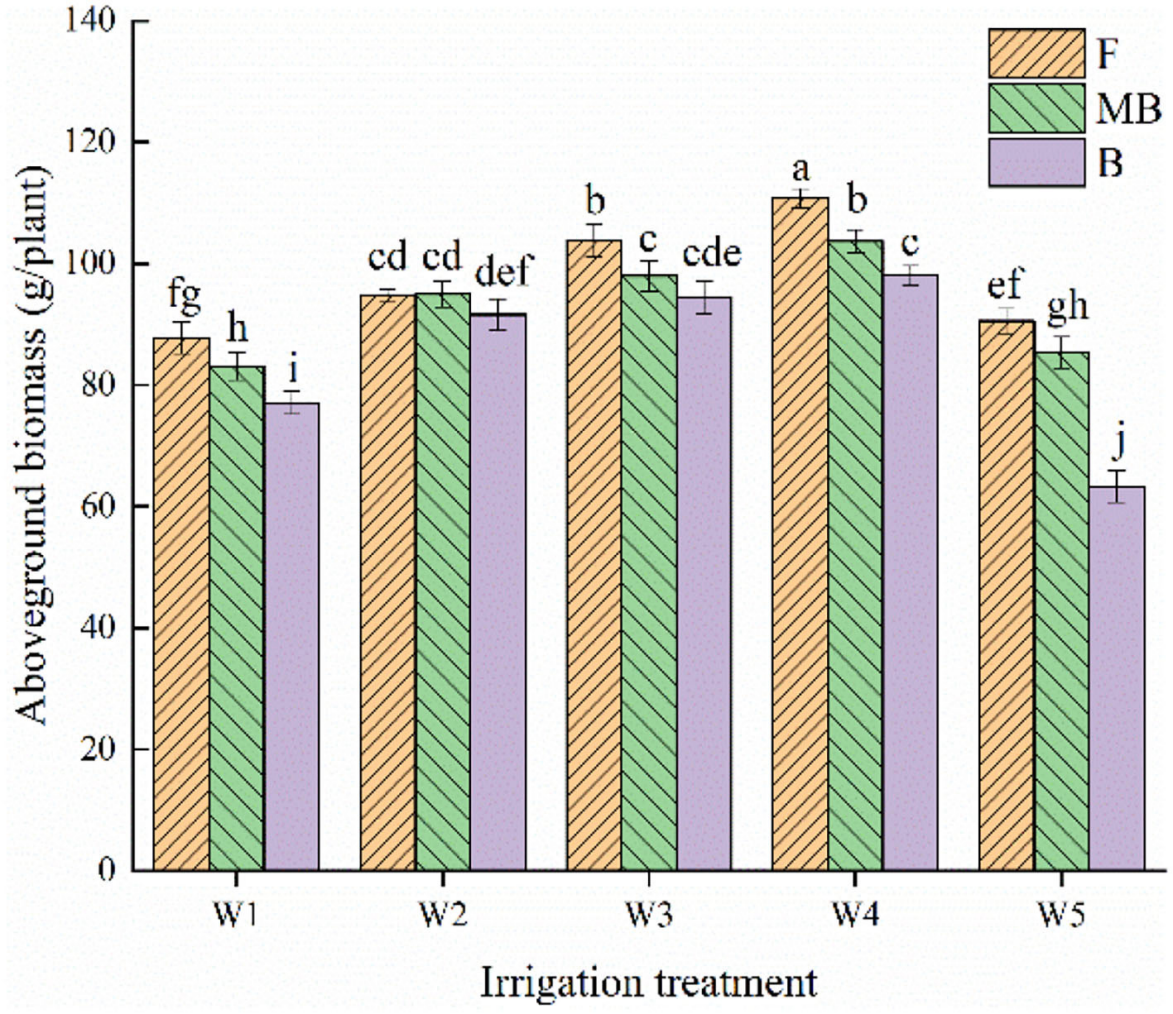
Effects of different irrigation treatments on aboveground biomass of *H. ammodendron*. F is freshwater treatment, MB is magnetized brackish water, B is brackish water. FW1, FW2, FW3, FW4, and FW5 represent irrigation amount of 81, 108, 135, 162, and 189 mm under freshwater treatment, respectively. MBW1, MBW2, MBW3, MBW4, and MBW5 represent irrigation amount of 81, 108, 135, 162, and 189 mm under magnetized brackish water treatment, respectively. BW1, BW2, BW3, BW4, and BW5 represent irrigation amount of 81, 108, 135, 162, and 189 mm under brackish water treatment, respectively. Data are mean value of the three replicates. Errors bars mean standard errors. Differences were determined using Duncan's multiple range test. Different letters above the bars indicate significant differences among treatments at *p* < 0.05.

Figure 1 shows the effects of irrigation water type and irrigation amount on the aboveground biomass of *H. ammodendron*. Irrigation water type and irrigation amount both significantly affected aboveground biomass (*p* < 0.05). The aboveground biomass increased with an increase in irrigation amount from W1 to W4 and decreased with increasing irrigation amount over the W4 level. The aboveground biomass of the fresh, magnetized brackish, and brackish water treatments all peaked under the W4 irrigation level, reaching 110.75, 103. 73, and 98.08 g/plant, respectively. The overall mean values of aboveground dry matter of *H. ammodendron* under fresh, magnetized brackish, and brackish water irrigation under different irrigation amounts were 97.54, 93.04, and 84.91 g/plant, respectively. The mean values of aboveground dry matter under magnetized brackish and brackish water irrigation were 4.61 and 12.96% lower than that under fresh water irrigation, respectively.

**Figure 2:**
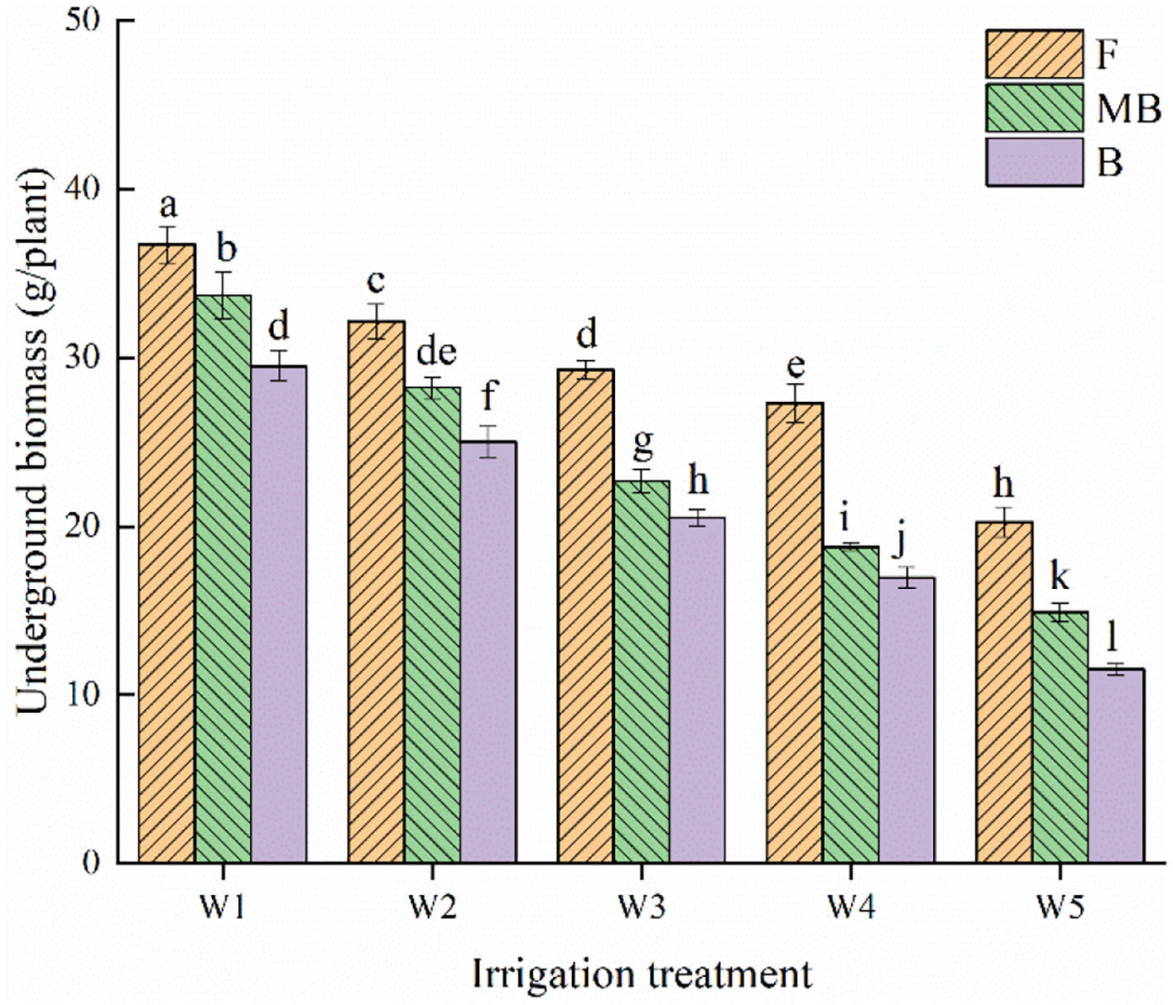
Effects of different irrigation treatments on underground biomass of *H. ammodendron*. F is freshwater treatment, MB is magnetized brackish water, B is brackish water. FW1, FW2, FW3, FW4, and FW5 represent irrigation amount of 81, 108, 135, 162, and 189 mm under freshwater treatment, respectively. MBW1, MBW2, MBW3, MBW4, and MBW5 represent irrigation amount of 81, 108, 135, 162, and 189 mm under magnetized brackish water treatment, respectively. BW1, BW2, BW3, BW4, and BW5 represent irrigation amount of 81, 108, 135, 162, and 189 mm under brackish water treatment, respectively. Data are mean value of the three replicates. Errors bars mean standard errors. Differences were determined using Duncan's multiple range test. Different letters above the bars indicate significant differences among treatments at *p* < 0.05.

Figure 2 shows the effect of different irrigation water types and irrigation amounts on the belowground biomass of *H. ammodendron*. Irrigation water type and irrigation amount both significantly affected the belowground biomass of *H. ammodendron* (*p* < 0.05). Within each respective irrigation type, the belowground biomass decreased with increasing irrigation amount, and the maximum value occurred under the W1 irrigation amount, reaching 36.72, 33.7, and 29.5 g/plant in the F, MB, and B treatments, respectively. Under the same irrigation amounts, the belowground dry matter of *H. ammodendron* was ordered F > MB > B. The mean values of the belowground dry matter of *H. ammodendron* under F, MB, and B irrigation under different irrigation amounts were 29.15, 23.66, and 20.70 g/plant, respectively. The mean values of belowground dry matter under MB and B irrigation were 18.82% and 28.97% lower than that under F irrigation, respectively.

**Figure 3:**
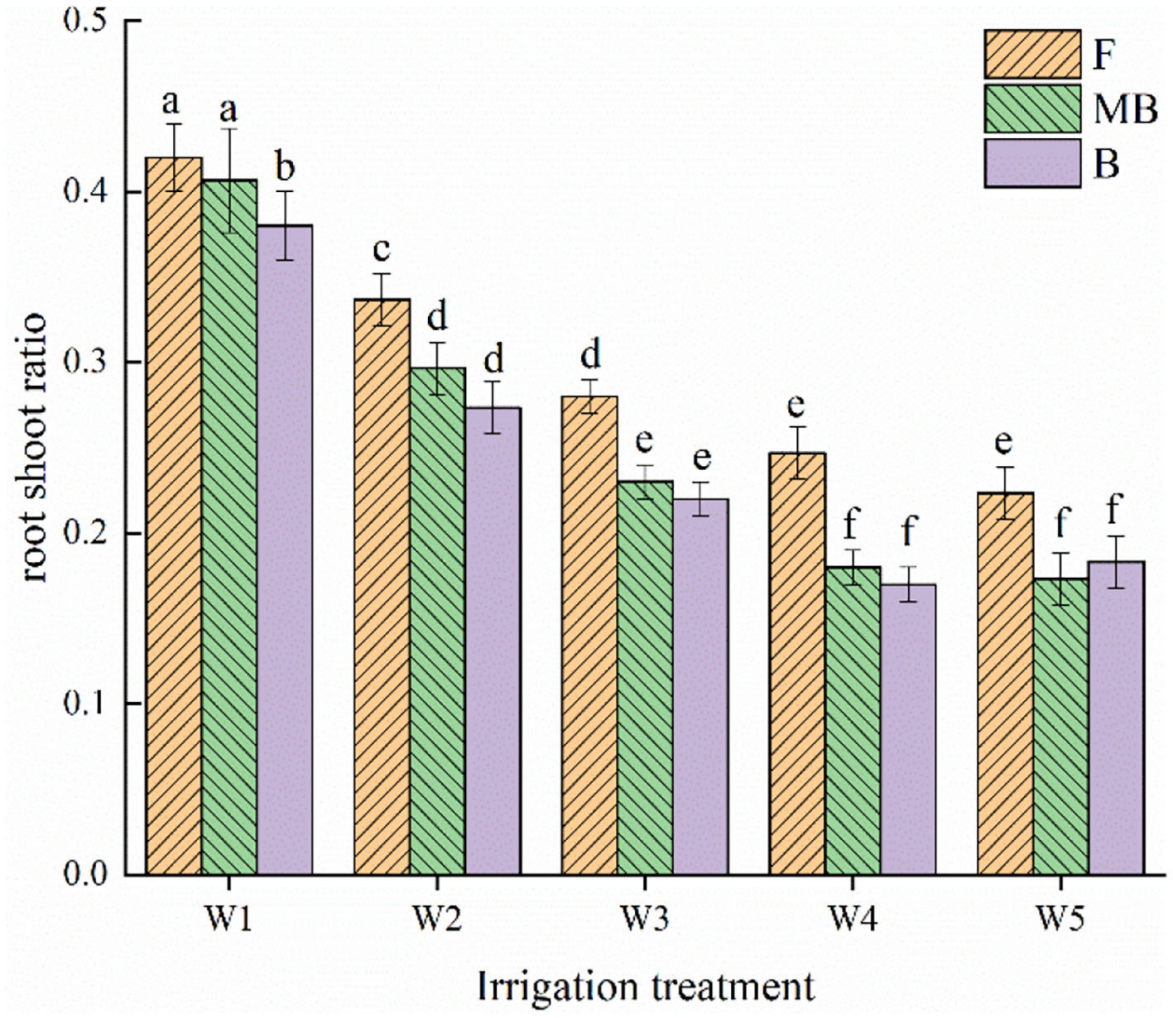
Effects of different irrigation treatments on root to shoot ratio of *H. ammodendron*. F is freshwater treatment, MB is magnetized brackish water, B is brackish water. FW1, FW2, FW3, FW4, and FW5 represent irrigation amount of 81, 108, 135, 162, and 189 mm under freshwater treatment, respectively. MBW1, MBW2, MBW3, MBW4, and MBW5 represent irrigation amount of 81, 108, 135, 162, and 189 mm under magnetized brackish water treatment, respectively. BW1, BW2, BW3, BW4, and BW5 represent irrigation amount of 81, 108, 135, 162, and 189 mm under brackish water treatment, respectively. Data are mean value of the three replicates. Errors bars mean standard errors. Differences were determined using Duncan's multiple range test. Different letters above the bars indicate significant differences among treatments at *p* < 0.05.

There were similar changes in the root shoot ratios of *H. ammodendron* for all treatments, as shown in Figure 3. Both irrigation water type and amount had significant effects on root shoot ratio (*p* < 0.05). Within each irrigation water type, the root shoot ratio decreased with increasing irrigation amount. The root shoot ratio was largest under the W1 treatment, reaching 0.42, 0.41, and 0.38 g/plant in the F, MB, and B treatments, respectively. Under the same irrigation amounts, the root shoot ratios were ordered F > MB > B. The mean values of the root shoot ratios for F, MB, and B irrigation treatments under different irrigation amounts were 0.30, 0.26, and 0.24 g/plant, respectively. The mean values of the root shoot ratio under MB and B irrigation were 14.57 and 19.21% lower than that under F irrigation.

**Figure 4:**
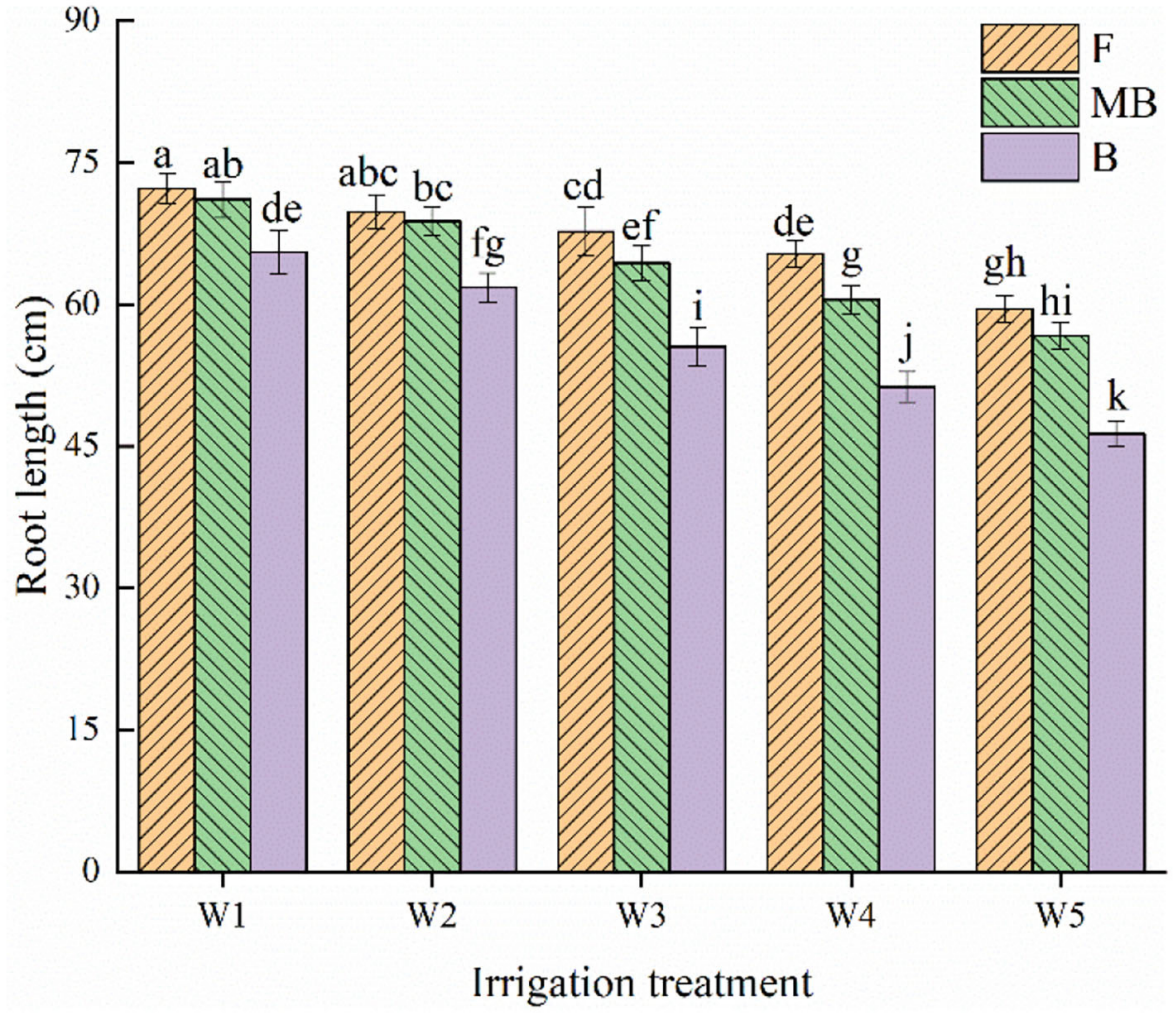
Effects of different irrigation treatments on root length of *H. ammodendron*. F is freshwater treatment, MB is magnetized brackish water, B is brackish water. FW1, FW2, FW3, FW4, and FW5 represent irrigation amount of 81, 108, 135, 162, and 189 mm under freshwater treatment, respectively. MBW1, MBW2, MBW3, MBW4, and MBW5 represent irrigation amount of 81, 108, 135, 162, and 189 mm under magnetized brackish water treatment, respectively. BW1, BW2, BW3, BW4, and BW5 represent irrigation amount of 81, 108, 135, 162, and 189 mm under brackish water treatment, respectively. Data are mean value of the three replicates. Errors bars mean standard errors. Differences were determined using Duncan's multiple range test. Different letters above the bars indicate significant differences among treatments at *p* < 0.05.

As for root length of *H. ammodendron*, the root length decreased with increasing irrigation amount (Figure 4). Irrigation water type and irrigation amount both significantly affected root length (*p* < 0.05). The root lengths under different water types were largest under the W1 treatment, reaching 72.29, 71.09, and 65.56 cm in the F, MB, and B treatments, respectively. Under the same irrigation amount, the overall mean values of root lengths under F, MB, and B irrigation under all irrigation amounts were 66.92, 64.28, and 54.15 cm, respectively. The mean values of root length under MB and B irrigation were 3.94 and 16.10% lower than that under F irrigation.

### Plant height, basal diameter, new shoot length, and crown width

**Figure 5:**
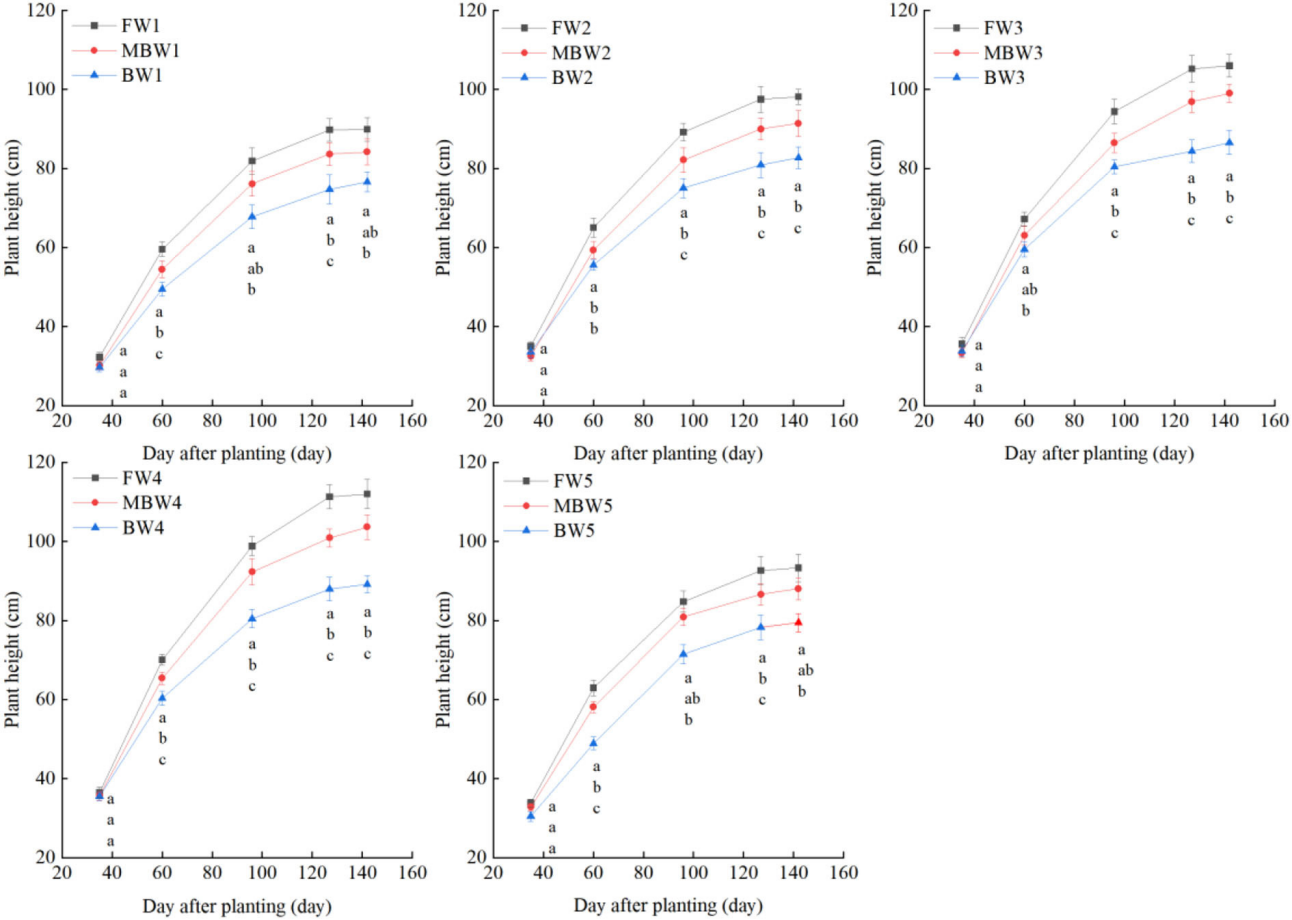
Effects of different irrigation treatments on plant height of *H. ammodendron*. FW1, FW2, FW3, FW4, and FW5 represent irrigation amount of 81, 108, 135, 162, and 189 mm under freshwater treatment, respectively. MBW1, MBW2, MBW3, MBW4, and MBW5 represent irrigation amount of 81, 108, 135, 162, and 189 mm under magnetized brackish water treatment, respectively. BW1, BW2, BW3, BW4, and BW5 represent irrigation amount of 81, 108, 135, 162, and 189 mm under brackish water treatment, respectively. Data are mean value of the three replicates. Errors bars mean standard errors. Differences were determined using Duncan's multiple range test. Different letters above the bars indicate significant differences among treatments at *p* < 0.05.

**Figure 6:**
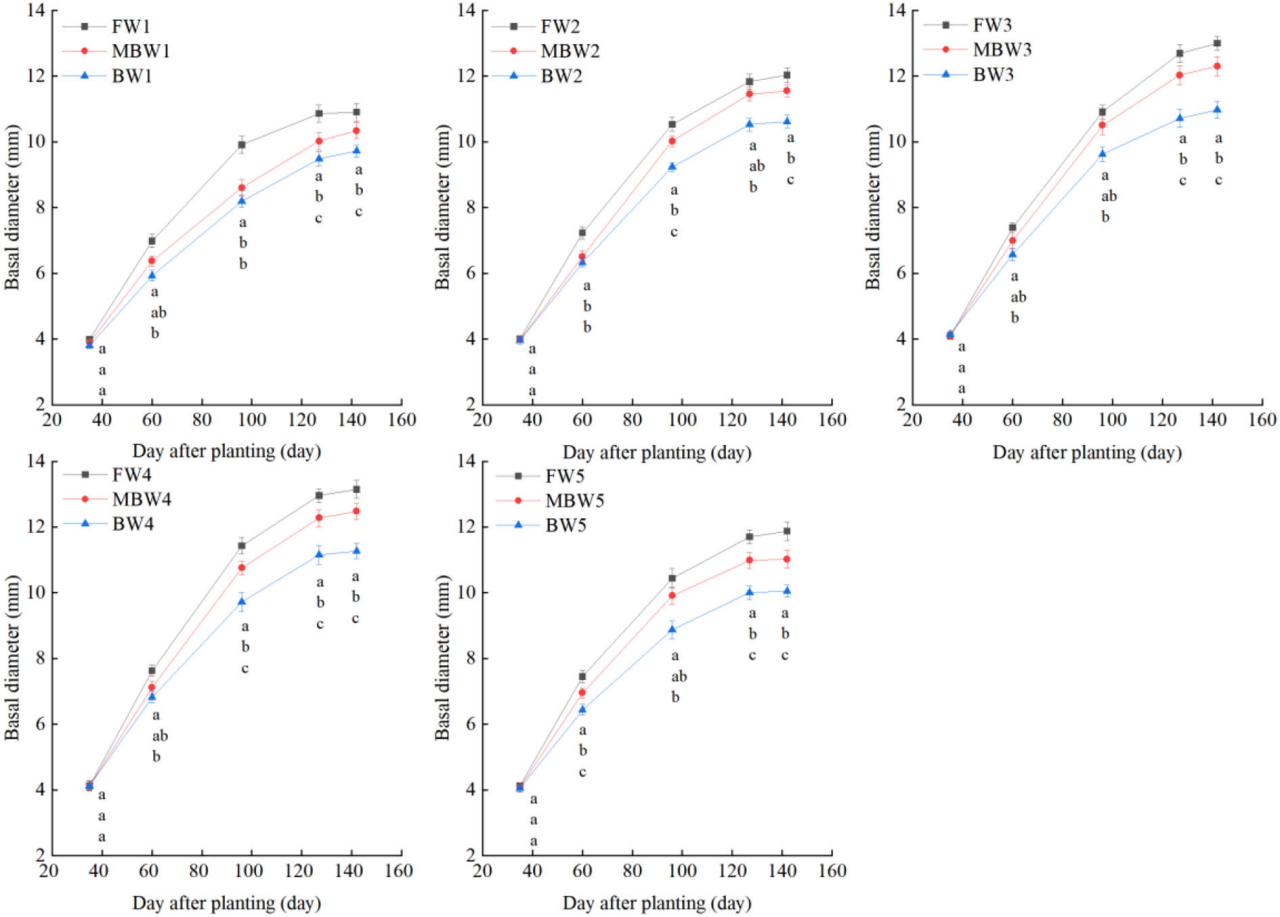
Effects of different irrigation treatments on basal diameter of *H. ammodendron*. FW1, FW2, FW3, FW4, and FW5 represent irrigation amount of 81, 108, 135, 162, and 189 mm under freshwater treatment, respectively. MBW1, MBW2, MBW3, MBW4, and MBW5 represent irrigation amount of 81, 108, 135, 162, and 189 mm under magnetized brackish water treatment, respectively. BW1, BW2, BW3, BW4, and BW5 represent irrigation amount of 81, 108, 135, 162, and 189 mm under brackish water treatment, respectively. Data are mean value of the three replicates. Errors bars mean standard errors. Differences were determined using Duncan's multiple range test. Different letters above the bars indicate significant differences among treatments at *p* < 0.05.

**Figure 7:**
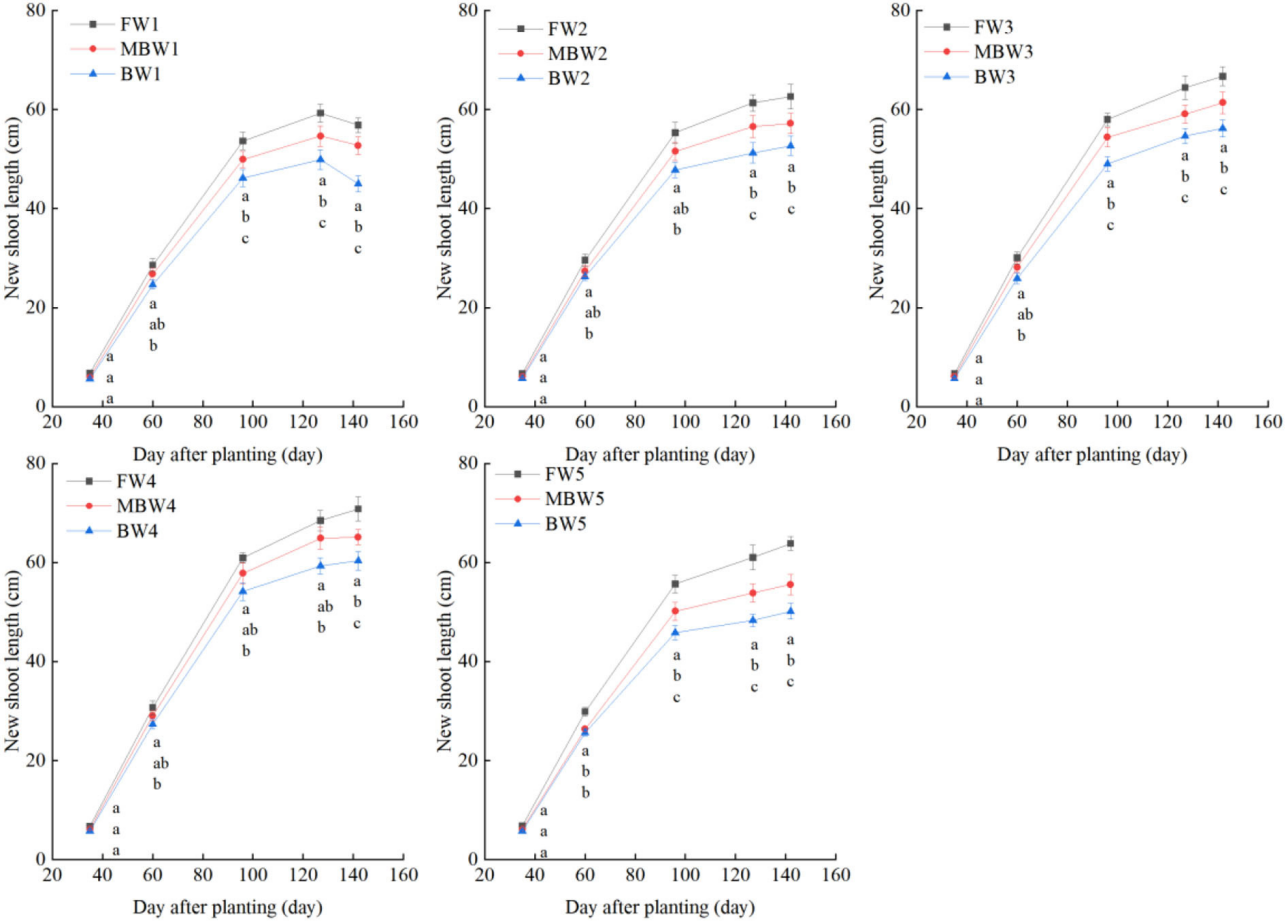
Effects of different irrigation treatments on new shoot length of *H. ammodendron*. FW1, FW2, FW3, FW4, and FW5 represent irrigation amount of 81, 108, 135, 162, and 189 mm under freshwater treatment, respectively. MBW1, MBW2, MBW3, MBW4, and MBW5 represent irrigation amount of 81, 108, 135, 162, and 189 mm under magnetized brackish water treatment, respectively. BW1, BW2, BW3, BW4, and BW5 represent irrigation amount of 81, 108, 135, 162, and 189 mm under brackish water treatment, respectively. Data are mean value of the three replicates. Errors bars mean standard errors. Differences were determined using Duncan's multiple range test. Different letters above the bars indicate significant differences among treatments at *p* < 0.05.

**Figure 8:**
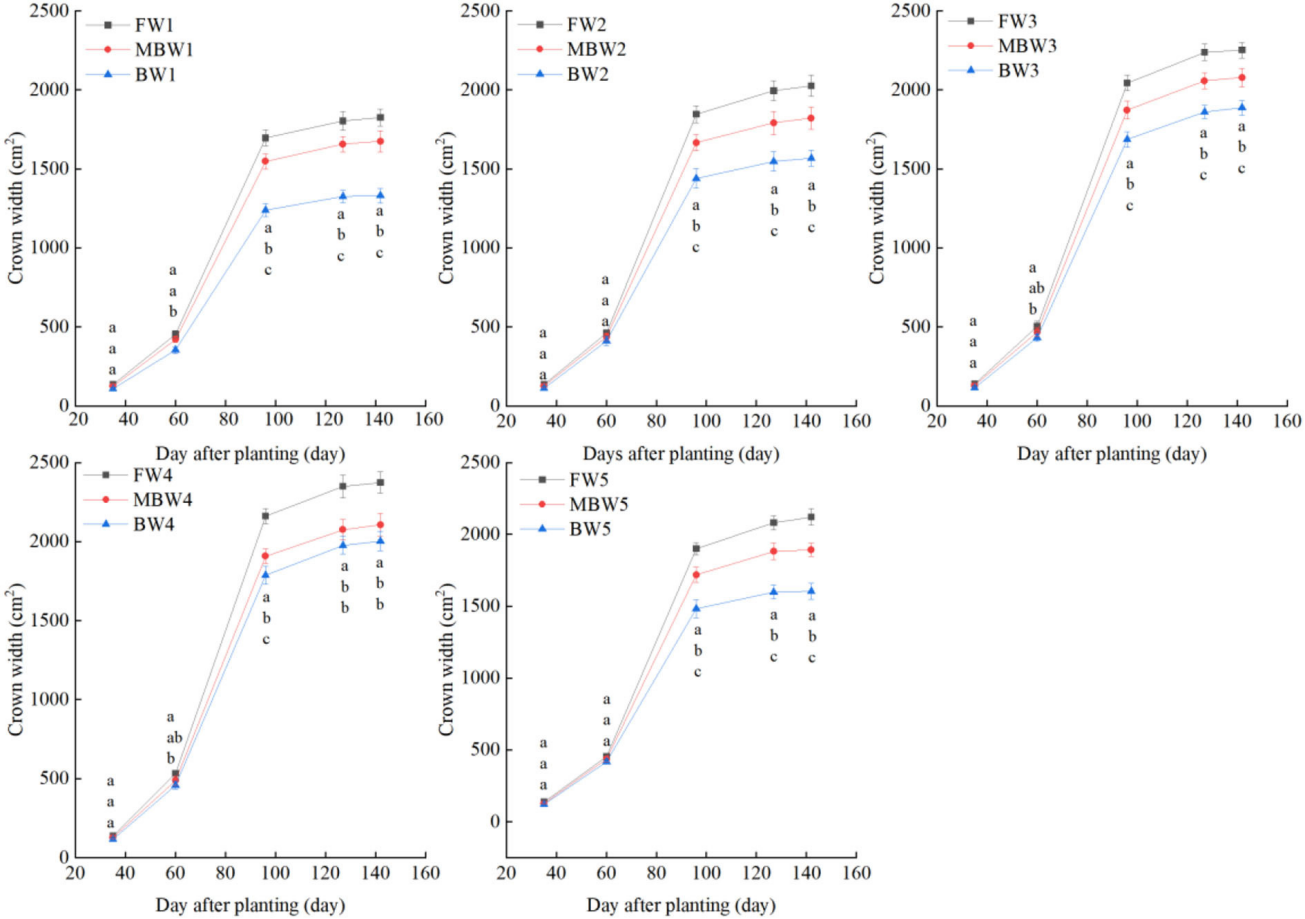
Effects of different irrigation treatments on crown width of *H. ammodendron*. FW1, FW2, FW3, FW4, and FW5 represent irrigation amount of 81, 108, 135, 162, and 189 mm under freshwater treatment, respectively. MBW1, MBW2, MBW3, MBW4, and MBW5 represent irrigation amount of 81, 108, 135, 162, and 189 mm under magnetized brackish water treatment, respectively. BW1, BW2, BW3, BW4, and BW5 represent irrigation amount of 81, 108, 135, 162, and 189 mm under brackish water treatment, respectively. Data are mean value of the three replicates. Errors bars mean standard errors. Differences were determined using Duncan's multiple range test. Different letters above the bars indicate significant differences among treatments at *p* < 0.05.

Figures 5–8 show the plant height, basal diameter, new shoot length, and crown width of *H. ammodendron* under different irrigation treatments. These growth indexes showed little change within 35 days of planting, but with the passage of time, the growth indexes gradually developed significant differences among different treatments (*p* < 0.05). Within each respective irrigation type, with increasing irrigation amount, the plant height, basal diameter, new shoot length, and crown width increased continuously, and these indicators all peaked with the W4 irrigation amount. These growth indexes were decreased when irrigation exceeded the W4 irrigation amount, suggesting that the W5 irrigation amount was not optimal for the growth of *H. ammodendron*. In addition, we found that under W1 irrigation, the new shoot length of *H. ammodendron* decreased 142 days after planting. This may have been because the W1 irrigation amount does not meet the growing conditions of *H. ammodendron* in the later growth stages, which will lead to the abscission of new branch growth in *H. ammodendron*. Under the same irrigation amounts, the *H. ammodendron* growth indexes of the treatments were ordered F > MB > B. Compared with fresh water irrigation, the plant height, basal diameter, new shoot length, and crown width of *H. ammodendron* under B irrigation decreased by 14.79–20.38%, 10.85–15.63%, 14.81–21.42%, and 15.68–27.12%, respectively. Compared with fresh water irrigation, plant height, basal diameter, shoot length, and crown width under MB water irrigation decreased by 5.64–7.49%, 3.95–7.14%, 7.21–12.99%, and 7.73–11.31%, respectively. This indicated that brackish water irrigation inhibited the growth of *H. ammodendron* seedlings and that the magnetization treatment alleviated that inhibition.

### Simulation of the *H. ammodendron* growth indexes

**Figure 9:**
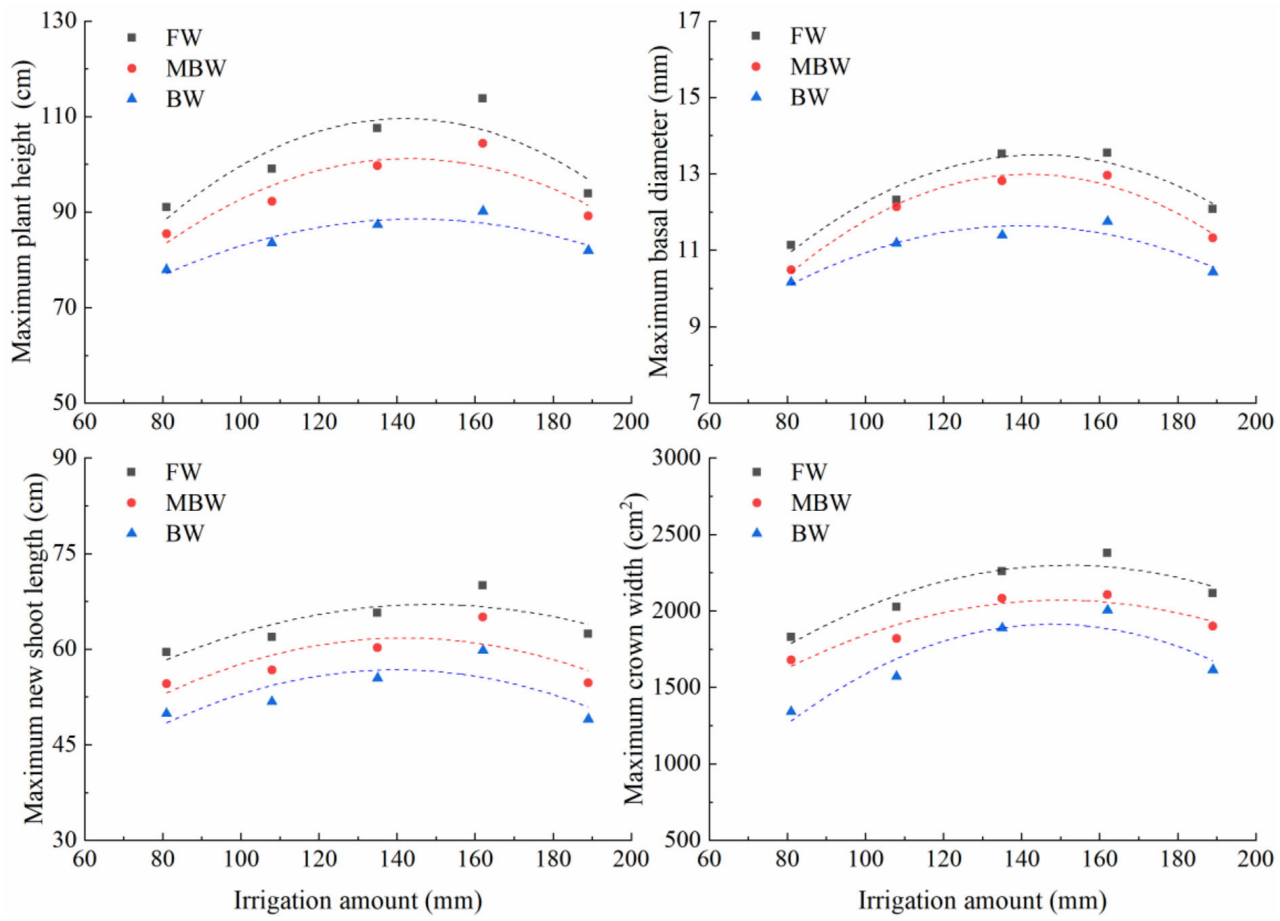
Relationships between maximum plant height, maximum basal diameter, maximum new shoot length, and maximum crown width and irrigation amount. FW is freshwater treatment, MBW is magnetized brackish water, BW is brackish water.

Plant height, basal diameter, new shoot length, and crown width are important indicators to reflect the growth state of *H. ammodendron*. The data of plant height, basal diameter, new shoot length, and crown width of *H. ammodendron* under different irrigation treatments were fitted by formulas (4), (5), (6), and (7). The fitting results are shown in Table 3. Within each respective irrigation type, irrigation amount had little effect on parameters *a*_1_, *a*_2_, *b*_1_, *b*_2_, *c*_1_, *c*_2_, *d*_1_, and *d*_2_. Therefore, to simplify the functions, it was assumed that the influence of irrigation amount on parameters *a*_1_, *a*_2_, *b*_1_, *b*_2_, *c*_1_, *c*_2_, *d*_1_, and *d*_2_ can be ignored, and these parameters were averaged. However, the maximum values of plant height, basal diameter, new shoot length, and crown width first increased and then decreased with increasing irrigation water amount (Figure 9). The maximum values of the growth indexes were all significantly affected by irrigation amount. The relationship between maximum plant height, maximum basal diameter, maximum shoot length, maximum crown width, and irrigation amount was expressed by the series of formulas in Table 4.

**Table 3:** Parameter values in logistic function of plant height, basal diameter, new shoot length, and crown width of *H. ammodendron*.

**Growth indexes**	**Irrigation treatment**	***H_*m*_***	***a*_1_**	***a*_2_**	***R* ^2^**	**Growth indexes**	***S_*m*_***	***c*_1_**	***c*_2_**	***R* ^2^**
Plant height	FW1	90.999	9.507	−0.0477	0.99	New shoot length	59.493	82.508	−0.0714	0.99
	FW2	99.010	9.655	−0.0480	0.99		61.910	79.622	−0.0704	0.99
	FW3	107.498	9.753	−0.0457	0.99		65.668	77.125	−0.0684	0.99
	FW4	113.705	10.002	−0.0454	0.99		69.949	75.365	−0.0667	0.99
	FW5	93.850	9.559	−0.0487	0.99		62.395	76.087	−0.0697	0.99
	Average	–	9.695	−0.047	-		–	78.141	−0.0693	–
	MBW1	85.431	8.904	−0.0455	0.99		54.535	99.090	−0.0753	0.99
	MBW2	92.205	8.960	−0.0458	0.99		56.747	95.031	−0.0739	0.99
	MBW3	99.708	8.995	−0.0444	0.99		60.201	95.740	−0.0730	0.99
	MBW4	104.396	9.132	−0.0450	0.99		65.027	93.544	−0.0711	0.99
	MBW5	89.149	8.536	−0.0456	0.99		54.700	95.768	−0.0743	0.99
	Average	–	8.905	−0.0453	–		–	95.834	−0.0735	–
	BW1	77.919	6.432	−0.0398	0.99		49.906	110.210	−0.0776	0.99
	BW2	83.568	6.561	−0.0426	0.99		51.761	108.018	−0.0779	0.99
	BW3	87.307	6.826	−0.0424	0.99		55.461	106.600	−0.0751	0.99
	BW4	90.144	6.924	−0.0434	0.99		59.783	105.320	−0.0741	0.99
	BW5	81.905	6.808	−0.0392	0.99		49.013	107.985	−0.0790	0.99
	Average	-	6.710	−0.0415	-		-	107.627	−0.0767	-
**Growth indexes**	**Irrigation treatment**	***D**_*m*_*	*b*_1_	*b*_2_	*R* ^2^	**Growth indexes**	***C**_*m*_*	*d*_1_	*d*_2_	*R* ^2^
Basal diameter	FW1	11.127	8.393	−0.0441	0.99	Crown Width	1828.551	850.408	−0.0950	0.99
	FW2	12.309	8.772	−0.0417	0.99		2024.913	889.548	−0.0939	0.99
	FW3	13.516	8.978	−0.0394	0.99		2259.644	902.068	−0.0934	0.99
	FW4	13.552	8.805	−0.0398	0.99		2377.806	933.252	−0.0941	0.99
	FW5	12.083	8.277	−0.0424	0.99		2116.270	878.564	−0.0923	0.99
	Average	-	8.645	−0.0415	-		-	890.768	−0.0937	-
	MBW1	10.482	7.072	−0.0410	0.99		1679.040	731.929	−0.0927	0.99
	MBW2	12.128	7.732	−0.0372	0.99		1819.210	782.718	−0.0929	0.99
	MBW3	12.809	7.836	−0.0373	0.99		2082.100	791.510	−0.0915	0.99
	MBW4	12.956	8.266	−0.0386	0.99		2105.071	809.144	−0.0924	0.99
	MBW5	11.315	7.637	−0.0417	0.99		1900.370	776.600	−0.0917	0.99
	Average	–	7.709	−0.0392	–		–	778.380	−0.0922	–
	BW1	10.160	5.469	−0.0339	0.99		1340.104	607.468	−0.0909	0.99
	BW2	11.177	6.394	−0.0355	0.99		1570.146	623.496	−0.0906	0.99
	BW3	11.390	6.333	−0.0364	0.99		1887.672	677.809	−0.0892	0.99
	BW4	11.749	6.558	−0.0364	0.99		2004.135	683.593	−0.0893	0.99
	BW5	10.433	5.605	−0.0365	0.99		1614.307	634.803	−0.0909	0.99
	Average	–	6.072	−0.0358	–		–	645.434	−0.0902	–

**Table 4:** Regression equations between maximum growth indexes and irrigation amounts.

**Regression equation**		***R* ^2^**	**Regression equation**		***R* ^2^**
$H_{Fm}=-0.0057*I^{2}+1.6103*I-4.4963$	(13)	0.79	$S_{Fm}=-0.0019*I^{2}+0.565*I+25.056$	(19)	0.71
$H_{MBm}=-0.0046*I^{2}+1.3123*I+7.3841$	(14)	0.82	$S_{MBm}=-0.0023*I^{2}+0.659*I+14.993$	(20)	0.61
$H_{Bm}=-0.0028*I^{2}+0.8125*I+29.782$	(15)	0.89	$S_{Bm}=-0.0024*I^{2}+0.6746*I+9.6058$	(21)	0.60
$D_{Fm}=-0.0006*I^{2}+0.1829*I+0.3056$	(16)	0.94	$C_{Fm}=-0.1012*I^{2}+30.75*I-38.759$	(22)	0.90
$D_{MBm}=-0.0007*I^{2}+0.1973*I-0.9897$	(17)	0.98	$C_{MBm}=-0.0911*I^{2}+27.292*I+25.596$	(23)	0.89
$D_{Bm}=-0.0004*I^{2}+0.1237*I+2.9961$	(18)	0.90	$C_{Bm}=-0.1412*I^{2}+41.755*I-1175$	(24)	0.87

Therefore, the equations for change in *H. ammodendron* plant height, basal diameter, new shoot, and crown width under fresh water, magnetized brackish water, and brackish water irrigation were, respectively:

$$\begin{array}{l} \{\begin{array}{l} H_{F}=\frac{-0.0057*I^{2}+1.6103*I-4.4963}{1+{9.695*e}^{-0.0471*t}}\;\left(25\right)\\ D_{F}=\frac{-0.0006*I^{2}+0.1829*I+0.3056}{1\;+\;8.645*e^{-0.0415*t}}\;\left(26\right)\\ S_{F}=\frac{-0.0019*I^{2}+0.565*I+25.056}{1\;+\;78.141*e^{-0.0693*t}}\;\left(27\right)\\ C_{F}=\frac{-0.1012*I^{2}+30.75*I-38.759}{1\;+\;890.768*e^{-0.0937*t}}\;\left(28\right) \end{array} \end{array}$$

$$\begin{array}{l} \{\begin{array}{l} H_{MB}=\frac{-0.0046*I^{2}+1.3123*I+7.3841}{1\;+\;8.536*e^{-0.0456*t}}\;\left(29\right)\\ D_{MB}=\frac{-0.0007*I^{2}+0.1973*I-0.9897}{1\;+\;7.709*e^{-0.0392*t}}\;\left(30\right)\\ S_{MB}=\frac{-0.0023*I^{2}+0.659*I+14.993}{1\;+\;95.834*e^{-0.0735*t}}\;\left(31\right)\\ C_{MB}=\frac{-0.0911*I^{2}+27.292*I+25.596}{1\;+\;778.380*e^{-0.0922*t}}\;\left(32\right) \end{array} \end{array}$$

$$\begin{array}{l} \{\begin{array}{l} H_{B}=\frac{-0.0028*I^{2}+0.8125*I+29.782\;}{1\;+\;6.710*e^{-0.0415*t}}\;\left(33\right)\\ D_{B}=\frac{-0.0004*I^{2}+0.1237*I+2.9961}{1\;+\;6.072*e^{-0.0358*t}}\;\left(34\right)\\ S_{B}=\frac{-0.0004*I^{2}+0.1237*I+2.9961}{1\;+\;107.627*e^{-0.0767*t}}\;\left(35\right)\\ C_{B}=\frac{-0.1412*I^{2}+41.755*I-1175}{1\;+\;645.434*e^{-0.0902*t}}\;\left(36\right) \end{array} \end{array}$$

**Figure 10:**
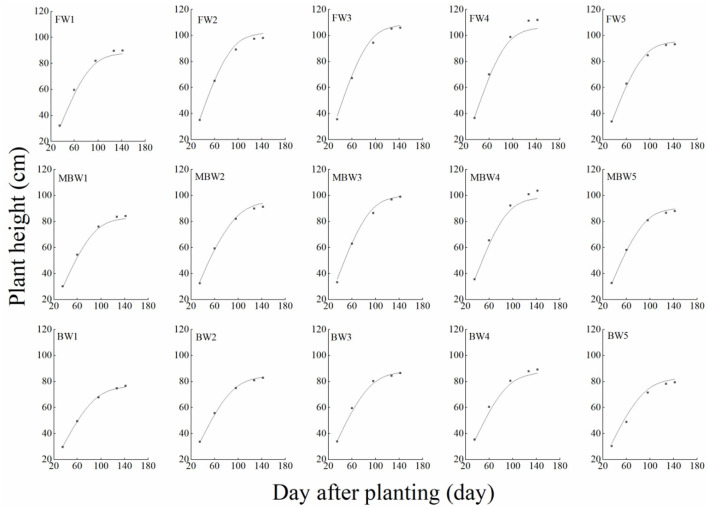
Comparison of simulated and measured plant heights of *H. ammodendron*. FW1, FW2, FW3, FW4, and FW5 represent irrigation amount of 81, 108, 135, 162, and 189 mm under freshwater treatment, respectively. MBW1, MBW2, MBW3, MBW4, and MBW5 represent irrigation amount of 81, 108, 135, 162, and 189 mm under magnetized brackish water treatment, respectively. BW1, BW2, BW3, BW4, and BW5 represent irrigation amount of 81, 108, 135, 162, and 189 mm under brackish water treatment, respectively.

**Figure 11:**
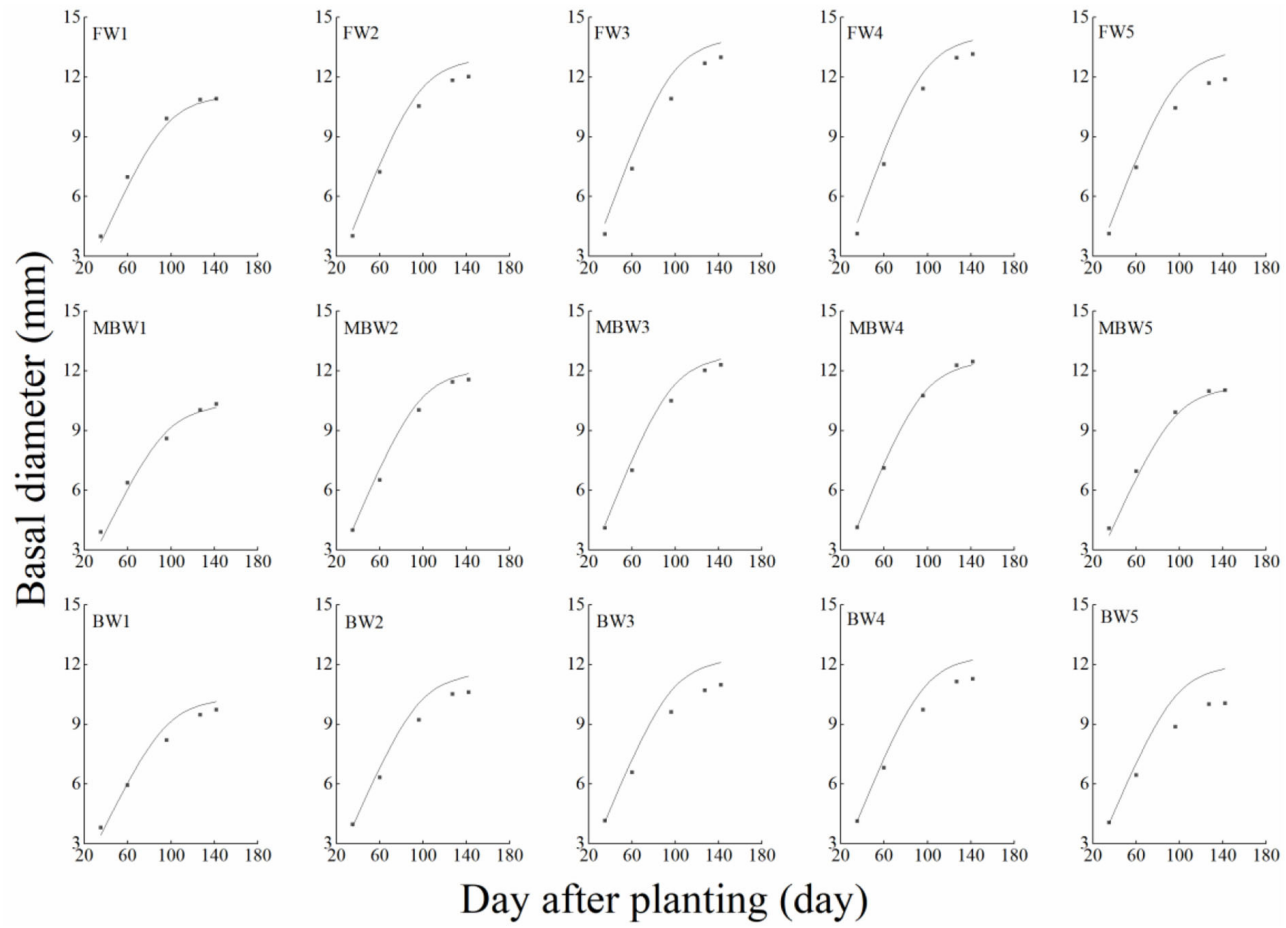
Comparison of simulated and measured basal diameters of *H. ammodendron*. FW1, FW2, FW3, FW4, and FW5 represent irrigation amount of 81, 108, 135, 162, and 189 mm under freshwater treatment, respectively. MBW1, MBW2, MBW3, MBW4, and MBW5 represent irrigation amount of 81, 108, 135, 162, and 189 mm under magnetized brackish water treatment, respectively. BW1, BW2, BW3, BW4, and BW5 represent irrigation amount of 81, 108, 135, 162, and 189 mm under brackish water treatment, respectively.

**Figure 12:**
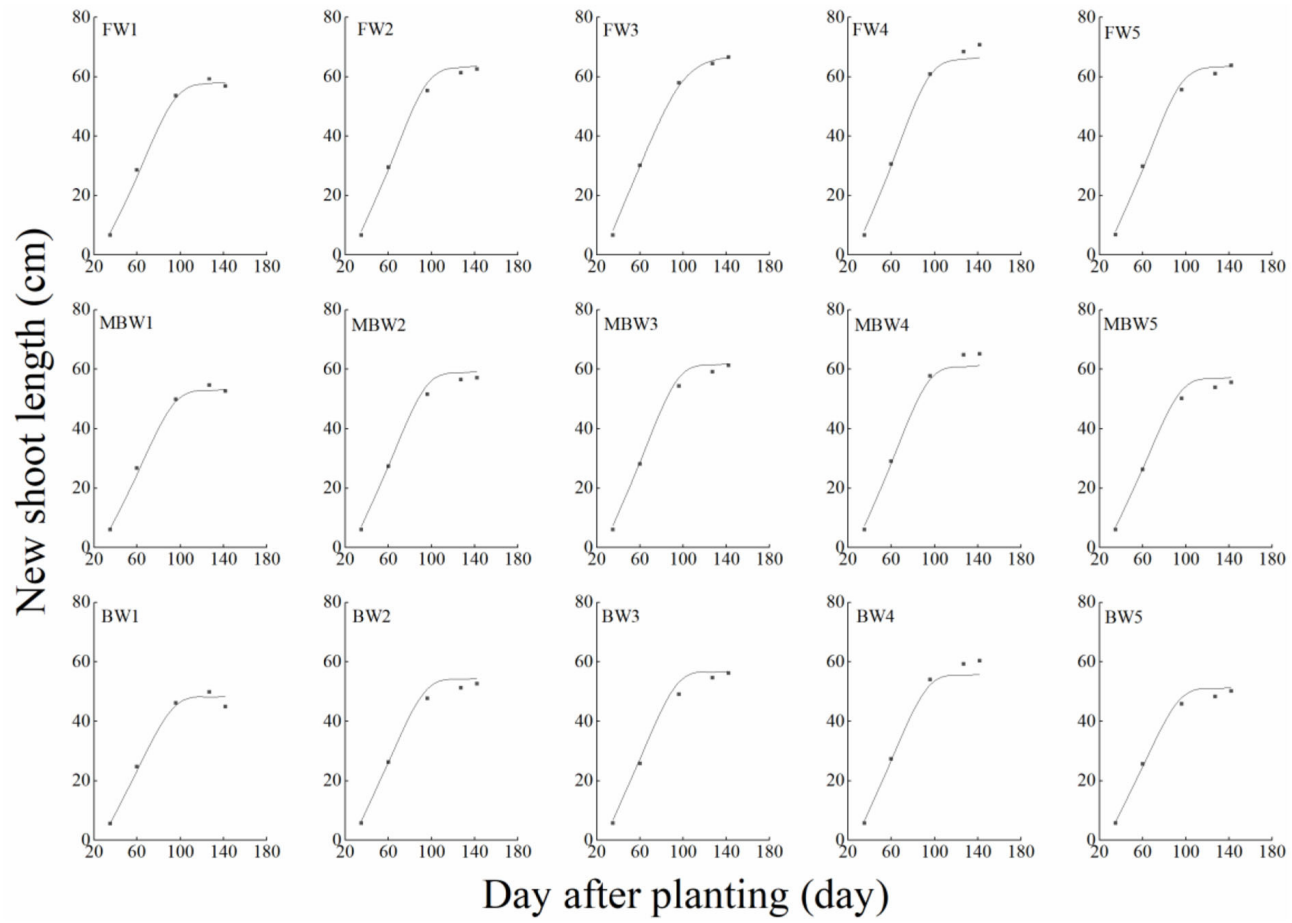
Comparison of simulated and measured new shoot lengths of *H. ammodendron*. FW1, FW2, FW3, FW4, and FW5 represent irrigation amount of 81, 108, 135, 162, and 189 mm under freshwater treatment, respectively. MBW1, MBW2, MBW3, MBW4, and MBW5 represent irrigation amount of 81, 108, 135, 162, and 189 mm under magnetized brackish water treatment, respectively. BW1, BW2, BW3, BW4, and BW5 represent irrigation amount of 81, 108, 135, 162, and 189 mm under brackish water treatment, respectively.

**Figure 13:**
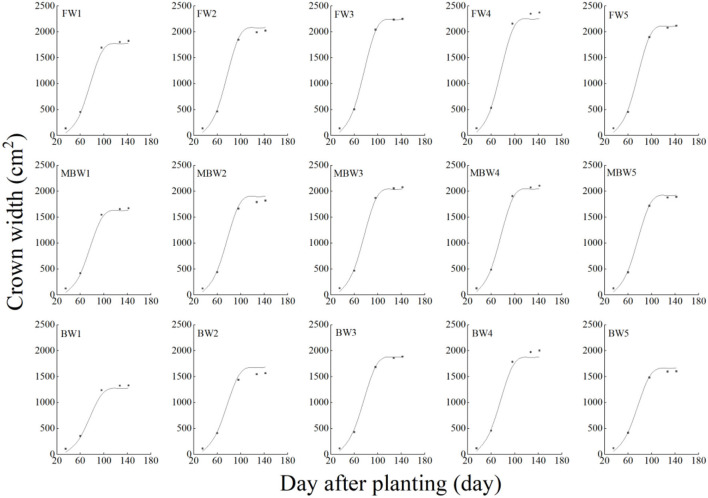
Comparison of simulated and measured crown widths of *H. ammodendron*. FW1, FW2, FW3, FW4, and FW5 represent irrigation amount of 81, 108, 135, 162, and 189 mm under freshwater treatment, respectively. MBW1, MBW2, MBW3, MBW4, and MBW5 represent irrigation amount of 81, 108, 135, 162, and 189 mm under magnetized brackish water treatment, respectively. BW1, BW2, BW3, BW4, and BW5 represent irrigation amount of 81, 108, 135, 162, and 189 mm under brackish water treatment, respectively.

In order to test the accuracy of the modified logistic functions, they were validated against the measured data, and the simulation results are shown in Figures 10–13. *R*^2^ and nRMSE were used to calculate the error between the simulated and measured values. The results showed that under different irrigation treatments, the ranges of nRMSE and *R*^2^, respectively, were 0.94–5.28% and 0.98–0.99 for plant height; 1.74–16.39% and 0.74–0.99 for basal diameter; 3.43–6.69% and 0.98–0.99 for shoot length; and 2.53–8.78% and 0.98–0.99 for crown width. This suggested that the established series of functions performed well in simulating plant height, basal diameter, new shoot length, and crown width.

### Water consumption and biomass water use efficiency

The effects of different irrigation treatments on water consumption and water use efficiency of *H. ammodendron* are shown in Table 5. When the irrigation amounts were the same, the water consumption under fresh water irrigation is significantly higher than that under brackish water irrigation (*p* < 0.05). Under W1–W4 irrigation amount, the water consumption of fresh water irrigation is greater than that of magnetized brackish water irrigation, but it is not significant (*p* > 0.05); Under W5 irrigation, the water consumption in fresh water irrigation is significantly greater than that of magnetized brackish water irrigation (*p* < 0.05). The water consumption under magnetized brackish water irrigation is higher than that under brackish water irrigation, but it is not significant (*p* > 0.05). Compared with brackish water irrigation, the water consumption of *H. ammodendron* under F and MB irrigation increased by 11.57–16.65% and 4.45–6.62%, respectively. Within each respective irrigation type, the water consumption of *H. ammodendron* increased with increasing irrigation amount. And there were no significant differences between treatments W3, W4, and W5 (*p* > 0.05).

**Table 5:** *H. ammodendron* evapotranspiration and biomass water use efficiency.

**Treatment**	**I (mm)**	**P (mm)**	**D (mm)**	**ΔW (mm)**	**ET (mm)**	**B (kg/hm^2^)**	**WUE_B_ (kg/ mm hm^2^)**
FW1	81	55.9	0	88.71 ± 7.1bc	225.61 ± 12.56fgh	3432.25 ± 56.01cd	15.24 ± 0.61a
MBW1	81	55.9	0	71.28 ± 7.13de	208.18 ± 13.26hi	3220.99 ± 71.87ef	15.02 ± 0.35ab
BW1	81	55.9	0	59.33 ± 2.97f	196.23 ± 12.24i	2938.49 ± 62.84h	15.00 ± 0.63ab
FW2	108	55.9	0	85.14 ± 9.37c	249.04 ± 12.12def	3499.00 ± 80.79c	14.06 ± 0.38c
MBW2	108	55.9	0	71.31 ± 5.7de	235.21 ± 12.76efg	3396.24 ± 78.57cd	14.45 ± 0.45bc
BW2	108	55.9	0	57.27 ± 3.44f	221.17 ± 13.99gh	3215.64 ± 65.92ef	14.12 ± 0.35c
FW3	135	55.9	0	88.79 ± 4.44bc	279.69 ± 14.15abc	3669.97 ± 73.08b	13.13 ± 0.41d
MBW3	135	55.9	0	70.93 ± 7.09de	261.83 ± 13.05cde	3326.69 ± 78.54de	12.72 ± 0.34def
BW3	135	55.9	0	59.78 ± 4.78f	250.68 ± 14.16def	3167.98 ± 98.47fg	12.65 ± 0.33def
FW4	162	55.9	22.51	96.7 ± 5.8ab	292.09 ± 18.69 ab	3805.86 ± 93.93a	13.05 ± 0.53de
MBW4	162	55.9	22.51	78.54 ± 3.93cd	273.93 ± 13.32bcd	3377.2 ± 84.11cd	12.34 ± 0.30ef
BW4	162	55.9	22.51	62.66 ± 4.39ef	258.05 ± 13.47cde	3170.86 ± 110.06fg	12.30 ± 0.23f
FW5	189	55.9	49.51	106.47 ± 10.65a	301.86 ± 18.62a	3054.57 ± 58.45gh	10.14 ± 0.44g
MBW5	189	55.9	49.51	80.51 ± 5.64cd	275.9 ± 15.49bcd	2764.21 ± 76.29i	10.03 ± 0.29g
BW5	189	55.9	49.51	63.38 ± 3.8ef	258.77 ± 13.58cde	2063.42 ± 59.94j	7.98 ± 0.19h
**Significance**							
Irrigation type				^***^	^***^	^***^	^***^
Irrigation amount				^***^	^***^	^***^	^***^

The data are all average ± standard error (the number of samples for each treatment is 3) in the table. I is irrigation, P is precipitation; D is drainage; ΔW is the change in water storage in the 1-m soil profile; ET is water consumption; B is biomass; WUE_B_ is biomass water use efficiency. Differences were determined using Duncan's multiple range test. Different letters within columns indicate significant differences among treatments at p < 0.05.

*** Indicates that it is significant at level of 0.001.

As for biomass water use efficiency, there was no significant difference (*p* > 0.05) in water use coefficient under the irrigation amount of W1, W2, and W3. Under W4 and W5 irrigation amount, there was no significant difference (*p* > 0.05) between fresh water and magnetized brackish water, and there was a significant difference (*p* < 0.05) between fresh water and brackish water. Within each respective irrigation type, with increasing irrigation amount, the biomass water use efficiency decreased with the increase of irrigation amount.

### Principal component analysis of the *H. ammodendron* growth indexes

A single growth index of *H. ammodendron* cannot fully reflect its growth status, so it is necessary to comprehensively analyze and evaluate various indexes of *H. ammodendron*. Principal component analysis (PCA) is a statistical method that transforms multi-index into a few comprehensive indexes by using the idea of dimension reduction (Xin et al., 2019; Wang X. K. et al., 2020; Bi et al., 2022). These comprehensive indicators retain most of the information of the original indicators (Shi et al., 2021; Tang et al., 2021). The Kaiser–Meyer–Olkin (KMO) value was 0.582 and Bartlett's sphericity test was *p* < 0.001, indicating that PCA could be performed (Hao et al., 2022). PCA was performed on 10 growth indexes (aboveground biomass, belowground biomass, root shoot ratio, root length, plant height, basal diameter, new shoot length, crown width, water consumption, and biomass water use efficiency) of *H. ammodendron*. The variance contribution analysis table, principal component load matrix, and eigenvector of each index were shown in Tables 6, 7. The percentage of the variance of the first principal component 1 (PC1) was 54.21%, and the percentage of the variance of the second principal component 2 (PC2) was 39.43%. The cumulative percentage of the variance of PC1 and PC2 was 93.64%. The PC1 and PC2 contained most of the information of the 10 indicators. The first principal component mainly included the crown width, new shoot length, basal diameter, plant height, water consumption, and aboveground biomass. The second principal component included the belowground biomass, root length, biomass water use efficiency, and root shoot ratio.

**Table 6:** Variance contribution analysis table.

**Component**	**Initial characteristics value**			**Extract square sum loading**		
	**Total**	**Variance/%**	**Cumulative/%**	**Total**	**Variance/%**	**Cumulative/%**
1	5.421	54.206	54.206	5.421	54.206	54.206
2	3.944	39.437	93.643	3.944	39.437	93.643
3	0.436	4.360	98.002			
4	0.098	0.980	98.982			
5	0.054	0.545	99.527			
6	0.019	0.195	99.722			
7	0.012	0.121	99.843			
8	0.011	0.112	99.954			
9	0.004	0.038	99.993			
10	0.001	0.007	100.000			

**Table 7:** Load matrix and eigenvector of each principal component.

**Index**	**Principal**		**Principal**	
	**component**		**component**	
	**load matrix**		**eigenvector**	
	**PC1**	**PC2**	**PC1**	**PC2**
Crown width	0.978	0.096	0.420	0.048
New shoot length	0.972	0.189	0.417	0.095
Basal diameter	0.958	0.246	0.411	0.124
Plant height	0.931	0.312	0.400	0.157
Water consumption	0.871	−0.407	0.374	−0.205
Aboveground biomass	0.812	0.413	0.349	0.208
Belowground biomass	−0.171	0.967	−0.073	0.487
Root length	−0.006	0.963	−0.003	0.485
Biomass water use efficiency	−0.225	0.922	−0.096	0.464
Root shoot ratio	−0.489	0.832	−0.210	0.419

Combining the standardized vector of the growth index and the eigenvector, the expression of the principal component was determined, and the comprehensive score was calculated:

The first principal component score was:

$$\begin{array}{l} Z_{1}=0.349*x_{1}-0.073*x_{2}-0.210*x_{3}-0.003*x_{4}\\ \;+0.400*x_{5}+0.411*x_{6}+0.417*x_{7}+0.420*x_{8}\\ \;+0.374*x_{9}-0.096*x_{10} \end{array}$$

The second principal component score was:

$$\begin{array}{l} Z_{2}=0.208*x_{1}+0.487*x_{2}+0.419*x_{3}+0.485*x_{4}\\ \;+0.157*x_{5}+0.124*x_{6}+0.095*x_{7}+0.048*x_{8}\\ \;-0.205*x_{9}+0.464*x_{10} \end{array}$$

The comprehensive evaluation formula was established as:

$$\begin{array}{l} Z=0.5421*Z_{1}+0.3944*Z_{2} \end{array}$$

where *Z*_1_ and *Z*_2_ are the first and second principal component scores, respectively, *Z* is the comprehensive score, and *x*_1_, *x*_2_, *x*_3_, *x*_4_, *x*_5_, *x*_6_, *x*_7_, *x*_8_, *x*_9_, and *x*_10_ are the standardized vector of aboveground biomass, belowground biomass, root shoot ratio, root length, plant height, basal diameter, new shoot length, crown width, water consumption, and biomass water use efficiency, respectively.

The comprehensive scores of the growth characteristics are shown in Table 8.

**Table 8:** Principal component scores and composite scores.

**Treatment**	**Principal component**			**Ranking**
	***Z*_1_**	***Z*_2_**	**Comprehensive score**	
FW1	−1.56	2.69	0.21	8
FW2	0.64	1.99	1.13	4
FW3	2.8	1.4	2.07	2
FW4	3.99	1.19	2.63	1
FW5	1.73	−1.47	0.36	6
MBW1	−2.78	2.11	−0.67	11
MBW2	−0.49	1.32	0.26	7
MBW3	1.47	0.13	0.85	5
MBW4	2.52	−0.58	1.14	3
MBW5	0.03	−2.49	−0.97	12
BW1	−4.52	0.95	−2.08	14
BW2	−2	0.11	−1.04	13
BW3	−0.34	−1.11	−0.62	10
BW4	0.66	−1.69	−0.31	9
BW5	−2.17	−4.55	−2.97	15

The treatments, ordered by their comprehensive scores from high to low, were FW4, FW3, MBW4, FW2, MBW3, FW5, MBW2, FW1, BW4, BW3, MBW1, MBW5, BW2, BW1, BW5. Thus, the three treatments with the highest scores were FW4, FW3, and MBW4, respectively. And the three treatments with the lowest scores were BW2, BW1, and BW5, respectively. Compared with brackish water irrigation, the comprehensive scores under magnetized brackish water irrigation were improved.

## Discussion

### Effect of different irrigation treatments on soil salt distribution

In this study in May, fresh water (F), magnetized brackish water (MB), and brackish water (B) all effectively leached salt from the soil. Within each respective irrigation type, the desalination rate increased with increasing irrigation amount. This indicated that salt leaching from the soil was directly dependent on irrigation amount, which was consistent with the study by Yuan et al. (2019) and Che et al. (2021). Within each respective irrigation amount, the salt washing effects of the three irrigation water types were ordered F > MB > B. Freshwater had the best washing effect because it had the lowest salinity, and therefore introduced the least salt into the soil during irrigation, but this was inconsistent with the previous findings (Zhou et al., 2016; Huang et al., 2021; Li et al., 2021). Notably, the salt washing effect of brackish water was enhanced after magnetization. This may have been due to the fact that, under the action of the magnetic field, the average distance between water molecules was increased and some hydrogen bonds became weak or even broke, and the large associated water molecule clusters were decomposed into free single and dimer molecules. Smaller water molecules are more likely to invade the small pores of the soil and carry more soil salt away with the water, increasing the convection and diffusion of soil salt and thus improving the soil salt washing efficiency. A similar study by Zhou et al. (2021) indicated that, compared with brackish water irrigation, magnetized brackish water irrigation increased the soil desalination rate by 29.2–50.4% in a cotton field. Wang Q. J. et al. (2020) also demonstrated that magnetized water had a better ability to dissolve and leach salt. Bu et al. (2010) and Zhang et al. (2013) reported that magnetized water irrigation improved the leaching of ${\text{SO}}_{4}^{2-}$, Cl^−^, and Na^+^ in soil.

Soil salt accumulation is mainly affected by environmental factors such as irrigation, evaporation, temperature, rainfall, and plant transpiration (Ding et al., 2016). In this study, the soil in the 0–100 cm depth range in September was in a salt accumulation state. This was due to the high temperatures and strong evaporation, resulting in the accumulation of salt in the shallower soil layers. Within each respective irrigation water type, soil salt accumulation increased with increasing irrigation water amount. This was because, as the irrigation amount increases, the salt carried by the water also increases accordingly. As the soil water evaporated, the salt in the deeper soil appeared to be carried to shallower soil layers with the water, which was consistent with previous research (Wei et al., 2021). Within the same irrigation amounts, the soil salt accumulation rate under the three irrigation water types was ordered MB > B > F. This was because the root growth indexes of *H. ammodendron* under magnetized brackish water irrigation were better than that under brackish water irrigation, which meant that *H. ammodendron* could enrich more salt when it absorbs more water under magnetized brackish water irrigation. In fact, soil salt content was affected by plant growth in the desert ecosystem. Some studies have shown that the salt content of the soil under shrubs is higher than that of the surrounding soil, which becomes the “salt island” effect. For example, Yin et al. (2012) studied the halophyte shrubs in the Tarim Basin, which showed that *Tamarix ramosissima, Halostachys caspica*, and *Halocnemum strobilaceum* all had “salt island” effects in varying degrees, and the salt island effect produced by the three enhanced the salt accumulation rate on the soil surface. Ge et al. (2007) found that after planting reed on saline-alkali soil, Na^+^ in soil increased significantly.

### Effects of irrigation treatments on growth indexes of *H. ammodendron*

Roots make up the majority of the biomass in desert plants. When water is scarce, plants prioritize allocating new biomass to building roots to enhance the uptake of soil water (Lu et al., 2021). Therefore, root length is very important for the establishment and growth of desert plants, especially seedlings. In this study, the root length of *H. ammodendron* increased with decreasing irrigation amount, indicating that *H. ammodendron* adapted to the decreased water supply through root elongation and growth, which was consistent with the results of Shan et al. (2008) and Lu et al. (2021). The root lengths of *H. ammodendron* when irrigated with magnetized brackish water and brackish water were shorter than when irrigated with fresh water, which may have been due to the salt accumulated in the soil from the high salinity brackish and magnetized brackish waters. Li et al. (2015) reported that in soil layers with high concentrations of salt, almost no desert plant roots could be found, and roots were mainly distributed in areas where the soil EC was less than 1 mS cm^−1^. In addition, the experimental results showed that magnetization treatment effectively reduced the inhibition effect of water salinity on root growth. This was similar to the experimental results of Zhao et al. (2022) who reported that magnetized water promoted root vigor and growth in winter wheat. The reasons underlying this phenomenon may have been twofold, on the one hand, magnetized water may promote plant metabolism, cell division, and differentiation, all of which would promote the growth of roots; on the other hand, magnetized water irrigation may enhance the leaching effect of soil salt and improve the growth environment for the *H. ammodendron* root system. Zhao et al. (2016) also reported that the numbers and lengths of rice seedlings roots irrigated with magnetized water increased by 21.74 and 20.62%, respectively, compared with unmagnetized water.

Changes in the root–shoot ratio reflect the distribution strategy of assimilated nutrients in different periods, which means the study of the plant root-to-shoot ratio is central to analyzing the distribution of plant assimilates (Schenk and Jackson, 2002). In this study, the root shoot ratio of *H. ammodendron* seedlings decreased gradually with increasing irrigation, which showed how the biomass allocation strategy of *H. ammodendron* seedlings changed according to water conditions. When the water was sufficient, the seedlings allocated more biomass to aboveground growth to maximize the capture of light energy and sustain plant consumption and growth. However, when there was a water shortage, seedlings allocated more resources to root growth to increase the water absorption potential so that they could sustain competitive growth rates. This was consistent with previous research (Xu et al., 2007a,b; Tian et al., 2014). In our study, compared with brackish water irrigation, the root shoot ratio under magnetized brackish water irrigation was larger, which benefits the survival of seedlings in arid environments. Similarly, Li et al. (2020) showed that under severe drought conditions, the root shoot ratio under magnetized water irrigation was 84.6% higher than that under tap water irrigation. Wei et al. (2020) showed that the root shoot ratio under magnetized water irrigation was 26.43% higher than that under non-magnetized water irrigation. Zhao et al. (2016) also reported that the root shoot ratio of rice seedlings was 20.18% higher after magnetization treatment.

Water is the most important factor determining the growth and development of desert plants, and changes in soil water greatly influence the ecological environment in desert areas (Wang et al., 2003). In this study, irrigation amount and type had no significant effect on plant height, stem diameter, new shoot length, and crown width in the first month after planting. This could be because the growth indexes of *H. ammodendron* seedlings were weakly affected by the external environment at this stage. This was consistent with the research of Liu et al. (2012) and Liu et al. (2018). Then *H. ammodendron* entered the fastest growing period of the whole year and was affected by the irrigation treatments. During this period, the shallow soil was supplemented by irrigation, so soil water content was plentiful, especially considering that the water demand of *H. ammodendron* seedlings is relatively small in this stage. At the end of the growing season, air temperature gradually increased, surface evaporation was strong, the water content in the shallow soil decreased, and environmental conditions for the growth of *H. ammodendron* seedlings deteriorated, which led to slowed growth. Within each respective irrigation type, the plant height, basal diameter, and new shoots of *H. ammodendron* increased continuously and then decreased with increasing irrigation amount. This was because, as the irrigation amount increased, the water absorbed by the *H. ammodendron* seedlings increased, which led to better growth. However, excessive irrigation led to high soil water content, which reduced soil permeability and inhibited the growth of *H. ammodendron* seedlings. In addition, we found that, under W1 irrigation, new shoots fell off at the end of the growing season. This was because the assimilating branches of *H. ammodendron* seedlings reduce water consumption by withering. Schulze et al. (1996) also showed that desert shrubs will use abscission to reduce water consumption during drought. Here, within the same irrigation amount, the plant height, basic diameter, and new shoots of *H. ammodendron* under the three irrigation water types were ordered F > MB > B. This indicated that brackish water inhibited the growth of *H. ammodendron*. Wang et al. (2010) and Chen et al. (2011) also showed that the physiological activity and growth rate of *H. ammodendron* decreased with increasing salinity of irrigation water. Zhang et al. (2020) showed that, compared with fresh water irrigation, the plant height, basal diameter, and new shoots of *H. ammodendron* under high salinity water irrigation decreased by 13.93–34.72, 4.76–66.15, and 27.54–63.71%, respectively. The effects of magnetized brackish water and brackish water on the growth of *H. ammodendron* were further compared, revealing that the magnetization treatment reduced the inhibiting effect of brackish water on the growth of the plant. This was because magnetized water irrigation promoted the growth of *H. ammodendron* roots, improved the absorption and utilization of water and nutrients, and indirectly affected the aboveground growth indexes. A similar study reported by Wang L. et al. (2019) also showed that magnetization treatment effectively alleviated the inhibitory effect of salt water irrigation on the growth of grape stems and leaves. Zhao et al. (2021) reported that magnetized water irrigation increased plant height, leaf area index, and dry matter accumulation of winter wheat.

In this study, we found that, within the same irrigation type, the irrigation amount had little effect on the empirical parameters *a*_1_, *a*_2_, *b*_1_, *b*_2_, *c*_1_, *c*_2_, *d*_1_, and *d*_2_ in the logistic functions, but had a significant effect on the maximum growth index values. Therefore, we averaged the empirical parameters, established the regression equations between irrigation amounts and the maximum values of each growth index, and then constructed a series of mathematical equations describing the plant height, basal diameter, new shoot length, and crown width of *H. ammodendron* under different irrigation amounts. Ma et al. (2013) and Wang et al. (2014) also indicated that irrigation amount had little impact on the empirical parameters in the wheat logistic function. The simulation results showed that it is reasonable to predict the growth in plant height, basal diameter, new shoot length, and crown width using the established functions.

### Effects of irrigation treatments on water consumption and biomass water use efficiency

In this study, water consumption of *H. ammodendron* increased with an increase in irrigation amount from W1 to W3. And when the irrigation amount exceeds the W3 level, the change in water consumption was not significant. When the irrigation amounts were the same, the water consumption under fresh water irrigation is significantly greater than that under brackish water irrigation and slightly greater than that under magnetized brackish water irrigation. This was because B and MB carried a large amount of salt, which resulted in the serious accumulation of salt in the soil and hindered the root system by absorbing water. Chen et al. (2011) and Hu et al. (2014) showed that brackish water and saline water irrigation significantly inhibited the physiological activity of *H. ammodendron*, and the water consumption decreased with increasing degree of mineralization of irrigation water. Wang et al. (2010) reported that water consumption of *H. ammodendron* during the growth period decreased by 22.1% when the salinity of irrigation water increased from 1 to 6 g L^−1^. Furthermore, the water consumption when irrigated with magnetized brackish water was slightly higher than that when using brackish water. This may be because magnetized brackish water enhanced the salt leaching effect, promoted root growth, and improved water absorption of *H. ammodendron*. These results were consistent with the findings of Zhou et al. (2021) and Zhao et al. (2022).

Water use efficiency reflects the ability of plants to effectively use water (Tennakoon and Milroy, 2003). In our study, over-irrigation reduced the WUE of *H. ammodendron*, while decreasing the irrigation amount increased WUE. There were two mechanisms driving this trend, one was because increasing irrigation amount can increase deep percolation, and the second was that *H. ammodendron* can resist drought by improving high water utilization efficiency during water deficits. This result agreed with many previous studies (Gong et al., 2006; Rouhi et al., 2007; Xu et al., 2007a,b; Yan et al., 2010; Yang et al., 2018). In addition, compared with fresh water irrigation, brackish water irrigation and magnetized brackish water irrigation slightly decreased the WUE. This is because brackish water and magnetized brackish water irrigation led to soil salt accumulation and inhibited root water absorption (Hu et al., 2014). Furthermore, compared with brackish water irrigation, magnetized brackish water irrigation had higher WUE because magnetized brackish water promoted root growth and enhanced water absorption. This result agreed with the earlier study by Zhao et al. (2022).

## Conclusion

In the current study, we proved the potential of magnetized brackish water as an eco-friendly technology for improving soil salt leaching and then for the growth of *H. ammodendron* seedlings in arid areas. Compared to unmagnetized brackish water, the magnetized brackish water significantly improved the effect of soil salt leaching, promoted root growth, and stimulated the growth of plant height, basal diameter, new shoot length, and crown width. In general, magnetized brackish water treatment slightly increased water consumption and water use efficiency. Furthermore, principal component analysis indicated that the comprehensive score of magnetized brackish water irrigation was higher than that of brackish water irrigation. The first three treatments with the highest scores are FW4, FW3, and MBW4, respectively. This showed that magnetized brackish water combined with an appropriate irrigation amount was helpful to optimize the growth of *H. ammodendron* seedlings based on freshwater saving. Therefore, we concluded that irrigation with magnetized brackish water was an effective method to ensure the establishment and growth of *H. ammodendron* seedlings in arid and water-deficient areas.

## Data availability statement

The raw data supporting the conclusions of this article will be made available by the authors, without undue reservation.

## Author contributions

YG: investigation, data curation, formal analysis, and writing—original draft. QW: conceptualization, writing—review and editing, and supervision. XZ, ZL, ML, JZ, and KW: investigation. All authors contributed to the article and approved the submitted version.

## Funding

This research is jointly supported by the National Natural Science Foundation of China (41830754, 52179042), Major science and technology projects of the XPCC (2021AA003-2) and Major Science and Technology Projects of Autonomous Region (2020A01003-3).

## Conflict of interest

The authors declare that the research was conducted in the absence of any commercial or financial relationships that could be construed as a potential conflict of interest.

## Publisher's note

All claims expressed in this article are solely those of the authors and do not necessarily represent those of their affiliated organizations, or those of the publisher, the editors and the reviewers. Any product that may be evaluated in this article, or claim that may be made by its manufacturer, is not guaranteed or endorsed by the publisher.
